# Spreading of a Prion Domain from Cell-to-Cell by Vesicular Transport in *Caenorhabditis elegans*

**DOI:** 10.1371/journal.pgen.1003351

**Published:** 2013-03-28

**Authors:** Carmen I. Nussbaum-Krammer, Kyung-Won Park, Liming Li, Ronald Melki, Richard I. Morimoto

**Affiliations:** 1 Department of Molecular Biosciences, Rice Institute for Biomedical Research, Northwestern University, Evanston, Illinois, United States of America; 2 Department of Molecular Pharmacology and Biological Chemistry, Feinberg School of Medicine, Northwestern University, Chicago, Illinois, United States of America; 3 Laboratoire d'Enzymologie et Biochimie Structurales, CNRS, Gif-sur-Yvette, France; The University of Arizona, United States of America

## Abstract

Prion proteins can adopt self-propagating alternative conformations that account for the infectious nature of transmissible spongiform encephalopathies (TSEs) and the epigenetic inheritance of certain traits in yeast. Recent evidence suggests a similar propagation of misfolded proteins in the spreading of pathology of neurodegenerative diseases including Alzheimer's or Parkinson's disease. Currently there is only a limited number of animal model systems available to study the mechanisms that underlie the cell-to-cell transmission of aggregation-prone proteins. Here, we have established a new metazoan model in *Caenorhabditis elegans* expressing the prion domain NM of the cytosolic yeast prion protein Sup35, in which aggregation and toxicity are dependent upon the length of oligopeptide repeats in the glutamine/asparagine (Q/N)-rich N-terminus. NM forms multiple classes of highly toxic aggregate species and co-localizes to autophagy-related vesicles that transport the prion domain from the site of expression to adjacent tissues. This is associated with a profound cell autonomous and cell non-autonomous disruption of mitochondrial integrity, embryonic and larval arrest, developmental delay, widespread tissue defects, and loss of organismal proteostasis. Our results reveal that the Sup35 prion domain exhibits prion-like properties when expressed in the multicellular organism *C. elegans* and adapts to different requirements for propagation that involve the autophagy-lysosome pathway to transmit cytosolic aggregation-prone proteins between tissues.

## Author Summary

Alzheimer's, Parkinson's, Huntington's, frontotemporal lobar degeneration (FTLD), amyotrophic lateral sclerosis (ALS), and prion diseases are all age-related, fatal neurodegenerative disorders. Hallmarks of these diseases include the expression of toxic protein species. The ability to spread and infect naive cells was thought to be limited to prions but has recently been observed for other disease-linked protein aggregates in tissue culture cells and transgenic mice. The underlying cellular pathways of this cell-to-cell transmission, however, remain elusive. We have developed a new prion model in the roundworm *Caenorhabditis elegans* and show that the appearance of aggregate species is associated with cellular toxicity, not only in the expressing cell but as well as in adjacent tissues. We monitored in real time the spreading of prion domains by autophagy-derived lysosomal vesicles from cell-to-cell. Given that autophagy and lysosomal degradation have a role in several neurodegenerative diseases, this cellular pathway might be the basis of amyloid infectivity in general.

## Introduction

Transmissible spongiform encephalopathies (TSEs) or prion diseases are fatal, age-related, and infectious neurodegenerative disorders that affect humans (e.g., Creutzfeldt-Jakob disease) and animals (e.g., scrapie in sheep and bovine spongiform encephalopathy in cattle) [1]. At the molecular level, prions propagate by recruitment and conversion of the soluble α-helix-rich cellular PrP^C^ into toxic aggregates of the pathological β-sheet-rich PrP^Sc^ isoform, via a mechanism described as seeded or nucleated polymerization [2]–[5]. The TSE agent is also infectious at the cellular level, where it transmits from cell-to-cell and infects naïve cells, both from within and outside the central nervous system [6], [7].

In yeast, prions can function as heritable epigenetic factors [8]–[12] upon forming an alternative self-propagating β-sheet-rich state from a soluble α-helix-rich fold. Yeast and mammalian prion determinants, however, do not share similarities in amino acid sequence, function, or subcellular localization. Yeast prions are naturally propagated within the cytosol from mother to daughter cells during cell division and require the disaggregase activity of the molecular chaperone Hsp104 to generate seeds and ensure dissemination [13]. In contrast, cell-surface localized mammalian prions are transmitted from cell-to-cell in terminally differentiated non-dividing cells. Sup35, like the majority of yeast prion proteins, contains a glutamine/asparagine (Q/N)-rich domain that confers the prion phenotype [14]. Although the mammalian prion protein PrP lacks this domain, other neurodegenerative disease proteins such as FUS (Fused in Sarcoma) and TDP-43 (TAR DNA-binding protein 43) have been shown to contain Q/N-rich prion-like domains [15]–[17].

There is increasing evidence that proteins closely associated with the neurodegenerative diseases Alzheimer's, Parkinson's, Huntington's, frontotemporal lobar degeneration (FTLD) and amyotrophic lateral sclerosis (ALS), exhibit prion-like properties [18]–[20]. Amyloid fibril assembly in general follows a nucleated polymerization reaction *in vitro* [9], and the addition of preformed fibrils or pathological brain extract seeds the polymerization of the corresponding monomeric protein in cell culture models or following injection into healthy mouse brains [24]–[31]. Furthermore, many proteins that form aggregates and fibrils exhibit cell non-autonomous effects and might spread among tissues within an organism [32]–[37]. The cellular processes and mechanisms that underlie cell-to-cell transmission of toxic protein species remain elusive in the current animal models that employ tissue culture cells and mice to investigate prion biology.

The nematode *Caenorhabditis elegans* has been widely used as a model system to investigate the biology of protein misfolding and toxicity [38]–[43], and has the advantage of transparent tissue types including muscle, intestinal, and neuronal tissue. We aimed to establish a new prion model system in this metazoan to examine the mechanisms of propagation of protein misfolding across tissues in a living organism. Since there are no known prions in *C. elegans*, and we wanted to avoid potential complications of infectious mammalian prions, we used the well-characterized cytosolic yeast prion protein Sup35 [8]. Here, we show that a cytosolic prion domain, NM, is highly toxic and can spread among tissues within the animal. The cell non-autonomous organismal toxicity of Sup35NM was associated with the accumulation of autophagy-derived vesicles, disruption of mitochondrial integrity, and the dynamic movement of the prion domain protein between tissues via autolysosomal vesicles.

## Results

### Properties of a *C. elegans* model for expression of the Sup35 prion domain

**Figure 1:**
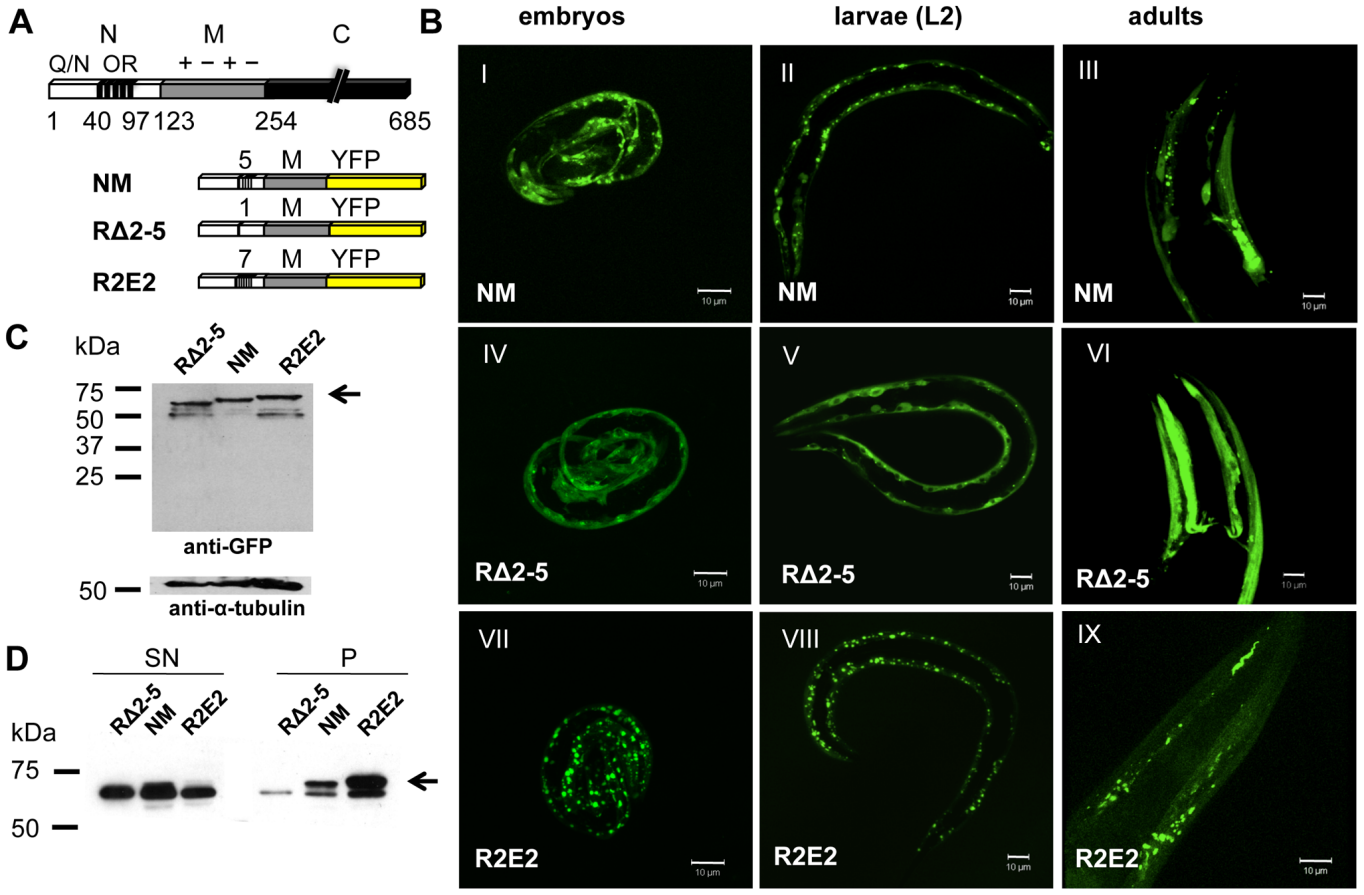
Sup35 prion domain aggregation is oligorepeat length–dependent. (A) Schematic representation of Sup35p and Sup35NM constructs. The yeast prion protein Sup35 comprises three regions, the aminoterminal (N), the middle (M), and the carboxyterminal (C) domain. N consists of a glutamine/asparagine (Q/N)-rich region (aa 1–40) and oligopeptide repeats (OR) (aa 41–97). The prion domain NM, NM with a deletion of oligorepeats number 2–5 (RΔ2-5), and NM with 2x extended oligorepeat number 2 (R2E2) were fused to YFP under the control of a BWM-specific promoter (*unc-54p*). (B) Collapsed confocal z-stack images of *C. elegans* lines stably expressing the indicated transgene. Pictures were taken at displayed stages during nematode development (embryo/L2 larvae/adult). Scale bar: 10 µm. (C) Total lysates of nematodes expressing RΔ2-5m::YFP, NMm::YFP, and R2E2m::YFP. Proteins were detected using an anti-GFP antibody. Anti-α-tubulin was used to demonstrate loading of comparable protein amounts. Arrows indicate full-length protein. Lower bands represent NM degradation products. YFP alone was not detected indicating that the tag was not cleaved off. (D) Detergent-solubility assay of lysates of nematodes expressing RΔ2-5m::YFP, NMm::YFP, and R2E2m::YFP. Proteins were detected using an anti-GFP antibody. SN = supernatant, P = pellet.

Three versions of Sup35NM, corresponding to the full-length wild-type domain, NM, a deletion of the oligorepeat region (RΔ2-5), and an expansion of the oligorepeats (R2E2), were fused to YFP (yellow fluorescent protein) (Figure 1A) and expressed under the control of the body wall muscle (BWM) cell specific (m) promoter *unc-54p*. These NM constructs were selected based on previous observations that deletion of four of the five oligorepeats of the prion domain (RΔ2-5) leads to a strong decrease in prion induction, while expansion of this region (R2E2) significantly increases spontaneous prion formation [44].

In *C. elegans* lines expressing approximately similar levels of the transgenes (Figure 1C), NMm::YFP aggregates appeared in early embryonic stages of development and persisted through all larval stages into adulthood (Figure 1B). The appearance of aggregates was strictly related to the length of the oligorepeats such that R2E2 formed aggregates more rapidly and to higher levels than NM, while deletion of the oligorepeats in RΔ2-5 resulted in expression of a mostly soluble and diffuse protein (Figure 1B, 1D). The fluorescent foci in NMm::YFP and R2E2m::YFP coincided with higher levels of detergent insoluble protein relative to RΔ2-5m::YFP (Figure 1D).

**Figure 2:**
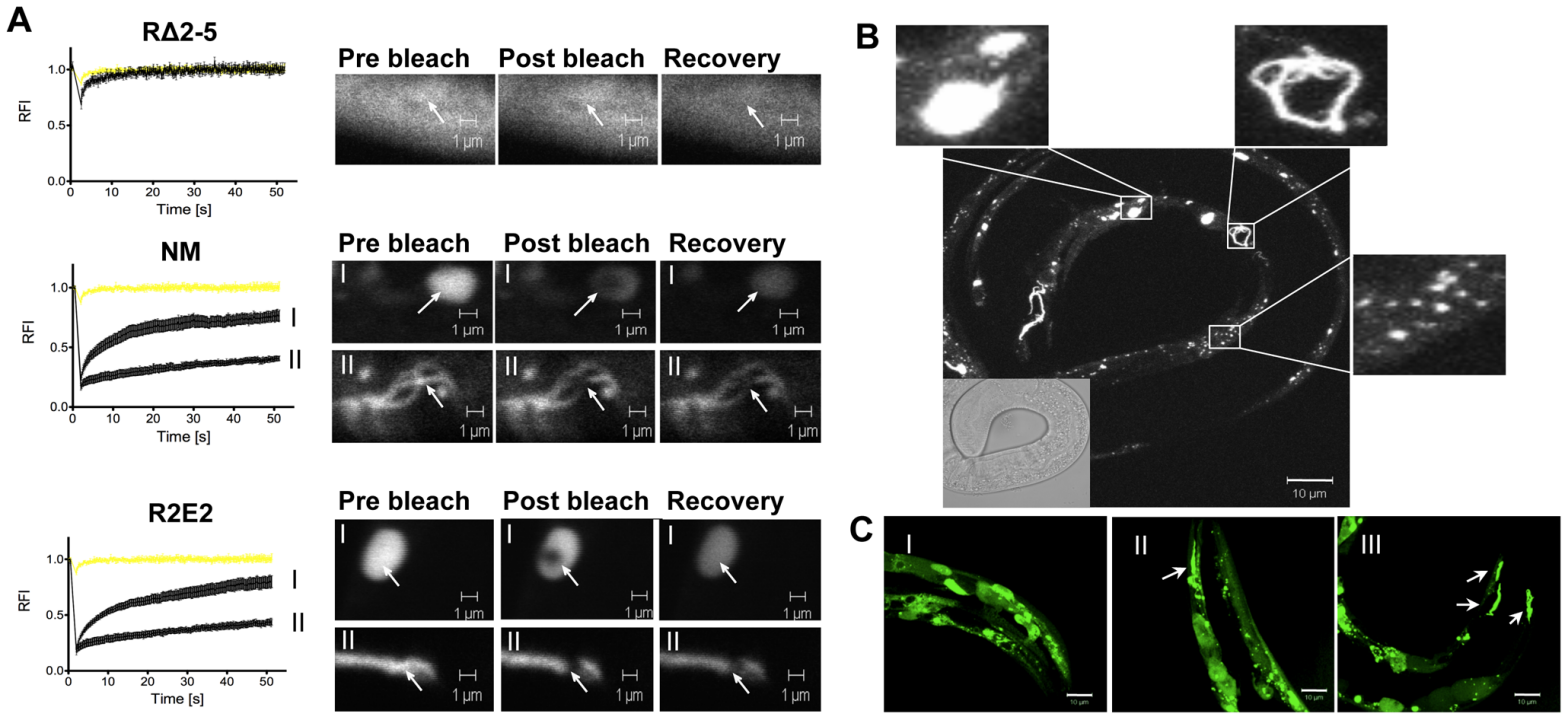
The prion domain forms biophysically and morphologically distinct aggregate types. (A) FRAP analysis of the indicated transgenic animals revealed mobile and immobile aggregate types that were grouped into two categories (see text). Aggregate mobility in animals expressing NMm::YFP and R2E2m::YFP correlated with a certain aggregate morphology. Roman numbers next to the FRAP graphs refer to representative foci (or diffuse staining pattern in case of RΔ2-5) that are shown to the right of each graph. Arrows indicate bleached ROI. The YFP only control is shown in yellow. RFI = relative fluorescence intensity in [%]. (B, C) Collapsed confocal z-stack images of R2E2m::YFP expressing transgenic *C. elegans*. (B) Aggregate shapes differed between muscle cells within one animal. (C) Aggregate types differed between the same cells of different animals. Arrows highlight fibril-like aggregates. Scale bars: 10 µm.

To further characterize the biochemical and biophysical properties of the NM aggregates, we employed the dynamic imaging method of fluorescence recovery after photobleaching (FRAP). The diffuse fluorescence observed in RΔ2-5m::YFP expressing animals was shown to correspond to highly mobile protein species by FRAP analysis, in addition to the infrequent appearance of foci that were too small to be assessed by FRAP (Figure 2A). In contrast, examination of NMm::YFP and R2E2m::YFP foci at high magnification revealed highly diverse shapes and sizes that can be described as long fibril-like species (∼10 µm), large (∼2 µm) round spherical structures, and small (∼0.1 µm) foci (Figure 2B, 2C). These foci did not exhibit any obvious patterns among the BWM cells and were randomly distributed. Moreover, each progeny descending from a single hermaphrodite exhibited a unique pattern of R2E2m::YFP foci (Figure 2C) suggesting an influence of the individual cellular environment on aggregate phenotypes.

FRAP analysis on animals expressing NM and R2E2 (Figure 2A) revealed foci ranging from immobile aggregates that exhibited no FRAP recovery to foci that rapidly recovered fluorescence and thus were comprised of mixed populations of slowly mobile protein species. These two biophysical states of prion domain aggregates were closely aligned with the distinct visual morphologies, in that every fibril-like aggregate tested was comprised of immobile species, and that spherical aggregates detected in both R2E2 and NM animals corresponded to mobile aggregates that showed recovery following photobleaching (Figure 2A).

### Cellular dysfunction associated with expression of the NM prion domain

**Figure 3:**
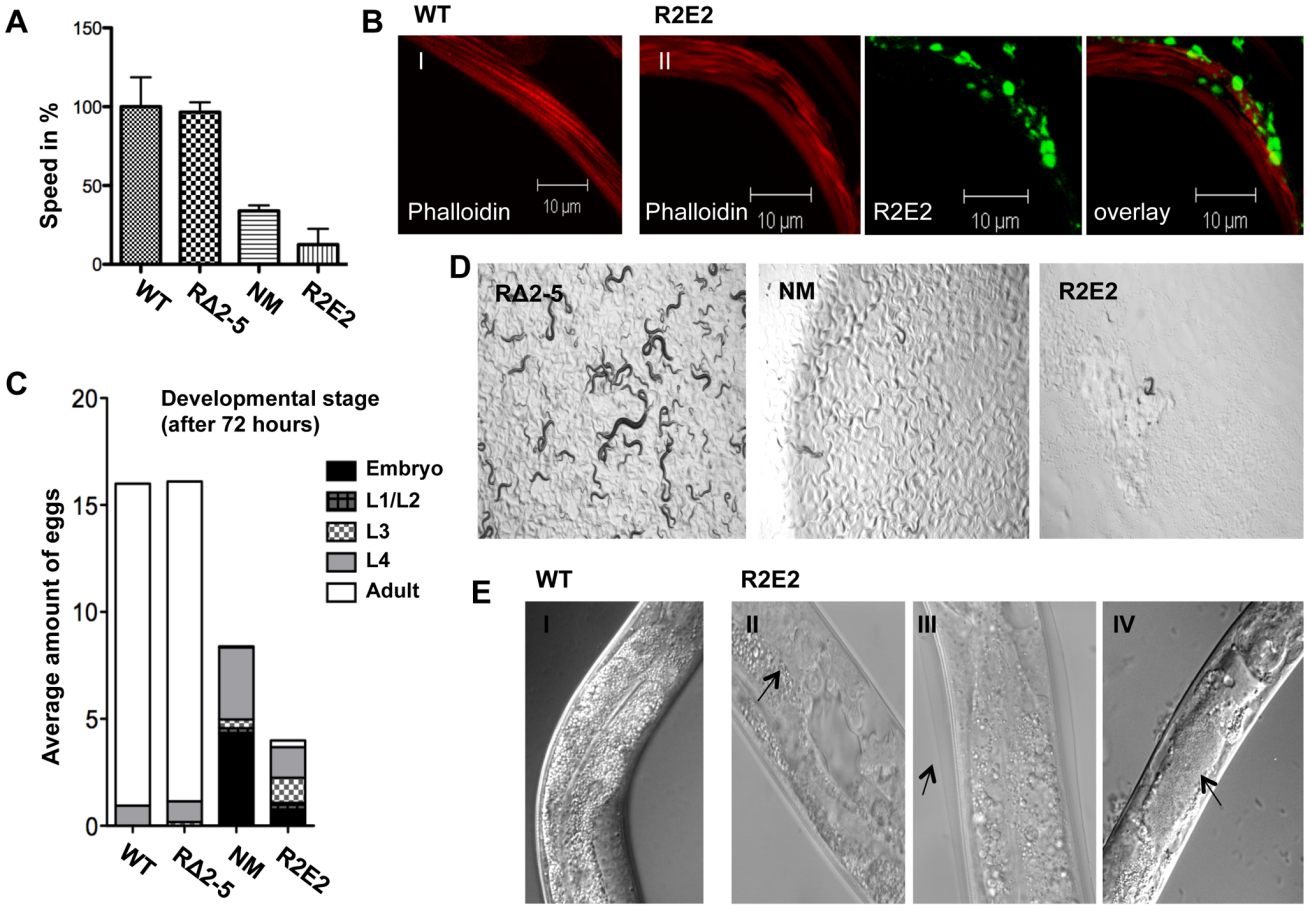
NMm::YFP and R2E2m::YFP aggregates are highly toxic. (A) Motility of 1-day-old adults was determined as speed in % relative to the wild-type N2 control. (B) Confocal images of control N2 (I) and R2E2m::YFP (II) expressing nematodes were stained with rhodamine-phalloidin to reveal actin fibers. Scale bars: 10 µm. (C) The average amount of eggs laid within 2.5 hours by the indicated *C. elegans* lines. The developmental stage of synchronized embryos was determined after 72 h (displayed as % of eggs laid). (D) 20 L1 larvae of *C. elegans* lines expressing the indicated transgene were grown at 20°C for 4 days before light micrographs were taken. Within this time wild-type N2 animals become adults and have progeny, some of which in turn would have developed into young adults, as seen with RΔ2-5, but not with NM and R2E2 transgenic animals. (E) DIC images of control (I), and R2E2m::YFP (II–IV) expressing animals. Arrows indicate abnormal gonad (II), old cuticle that has failed to shed and remains attached indicating molting defects (III) and disrupted intestinal cells bordering the distended lumen (IV).

R2E2m::YFP animals exhibited a severe reduction in motility relative to wild-type N2 or RΔ2-5m::YFP animals (Figure 3A; Video S1, S2, S3) that was associated with a disruption of muscle ultrastructure revealed by rhodamine-phalloidin staining of myofilaments (Figure 3B). Moreover, nearly all of the R2E2m::YFP and NMm::YFP adults exhibited developmental delay and reduced fecundity, with R2E2m::RFP adults being often sterile (Ste) (Figure 3C, 3D). Whereas adult N2 wild-type and RΔ2-5 animals lay approximately 16 eggs within a 2.5 hour period, NM and R2E2 animals laid 8.5 and 4 eggs, respectively (Figure 3C). Furthermore, only 8% of R2E2m::YFP embryos and 1% of NMm::YFP embryos attained adulthood over a three day period at 20°C, relative to >93% achieving adulthood for wild-type N2 or RΔ2-5m::YFP embryos (Figure 3C). The slightly higher fraction of adult R2E2 animals detected after 72 hours is due to a more severe egg laying defect (Egl) of these animals. R2E2 animals often retained their eggs due to dysfunction of the vulva muscle leading to embryos being laid at later time points of development. Consequently, eggs laid by R2E2 animals tended to be older than corresponding NM, RΔ2-5, and wild-type N2 embryos that are deposited at the same time. NM animals exhibited a more severe embryonic lethal phenotype (Emb) than R2E2, while the latter animals exhibiting increased sterility (Ste) and producing fewer total progeny (Figure 3C). Animals that reached the L4 state of development after 72 hours became adult animals on the next day, whereas younger larvae were more likely to arrest in development (Figure 3D, Video S1, S2, S3, data not shown). In summary, while the populations of NM and R2E2 animals differed in their distribution among developmental states after 72 hours, expression of the highly aggregation-prone R2E2 resulted in a more severe toxic phenotype than NM (Figure 3C, 3D; Video S1, S2, S3).

Another characteristic of R2E2m::YFP expressing animals was a plethora of morphological defects that included reduced size (Sma), vacuolation (Vac), defective molting (Mlt), clear appearance (Clr), and disrupted gonadal and intestinal morphology (Figure 3E, and data not shown). Such defects affecting other tissues were observed to a lesser extent in NMm::YFP animals, and not detected in RΔ2-5m::YFP lines or in *C. elegans* lines expressing other aggregation-prone proteins [38], [39], [41]–[43] (data not shown).

**Figure 4:**
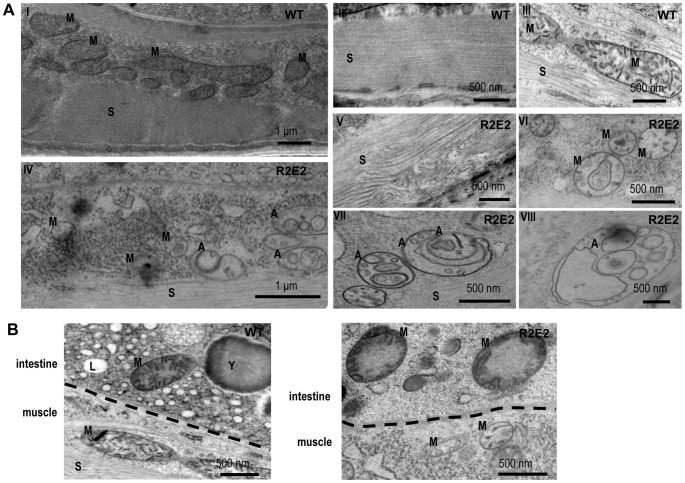
Transmission Electron Microscope (TEM) analysis reveal that mitochondrial defects and induction of autophagy are prominent features of prion domain–induced pathology. (A) Transmission Electron Microscope (TEM) images of control (I–III) and R2E2m::YFP (IV–VIII) adult animals show intact muscle sarcomeres (II) and mitochondrial morphology (III) in wild-type N2s, while R2E2m::YFP animals suffer from a disruption of sarcomeric structure (V) and mitochondrial morphology (VI), and an accumulation of autophagosomes (VII), as well as complex autophagy-related endo/lysosomal vesicles (VIII). (B) TEM analysis of R2E2m::YFP revealed disrupted mitochondrial integrity in neighboring intestinal cells in contrast to control N2 animals. A: autophagy-related vesicles; M: mitochondria; S: sarcomere; Y: yolk granules; L: lipid droplets.

The NM-dependent cellular defects were examined in more detail using transmission electron microscopy (TEM). Compared to wild-type N2 animals, the muscle cells of R2E2 expressing animals exhibited disrupted sarcomeres, fragmented mitochondria containing a drastically reduced number of cristae, and a complex array of double and single membrane bound organelles (Figure 4A). These vesicular structures are a hallmark of autophagy. Surprisingly, the cellular pathology observed in R2E2 expressing animals was not restricted to BWM cells and was also observed in other tissues in which R2E2 was not expressed. For example, intestinal cells that did not express R2E2 exhibited mitochondrial fragmentation with loss of cristae, and reduced levels of yolk and lipid droplets (Figure 4B).

These studies show that the number of oligorepeats in the prion domain directs the toxicity that results in multiple organismal phenotypes that extend beyond the primary tissue of NM expression.

### The prion domain is targeted to lysosomal degradation

To examine whether the induction of autophagy is a secondary cellular response due to damage of essential components like mitochondria, or if the prion domain is directly targeted by the autophagy-lysosome pathway (ALP), we employed *C. elegans* lines expressing markers of specific membraneous organelles. As the available markers for *C. elegans* are tagged with green fluorescent protein (GFP), we generated a *C. elegans* line expressing R2E2 tagged with red fluorescent protein (RFP) under the control of the *myo-3* promoter for BWM cell specific expression (R2E2m::RFP). LGG-1::GFP transgenic animals that express the ortholog of the autophagosome marker LC3 (in mammals) or ATG8 (in yeast) were used to monitor autophagic vesicles [45].

**Figure 5:**
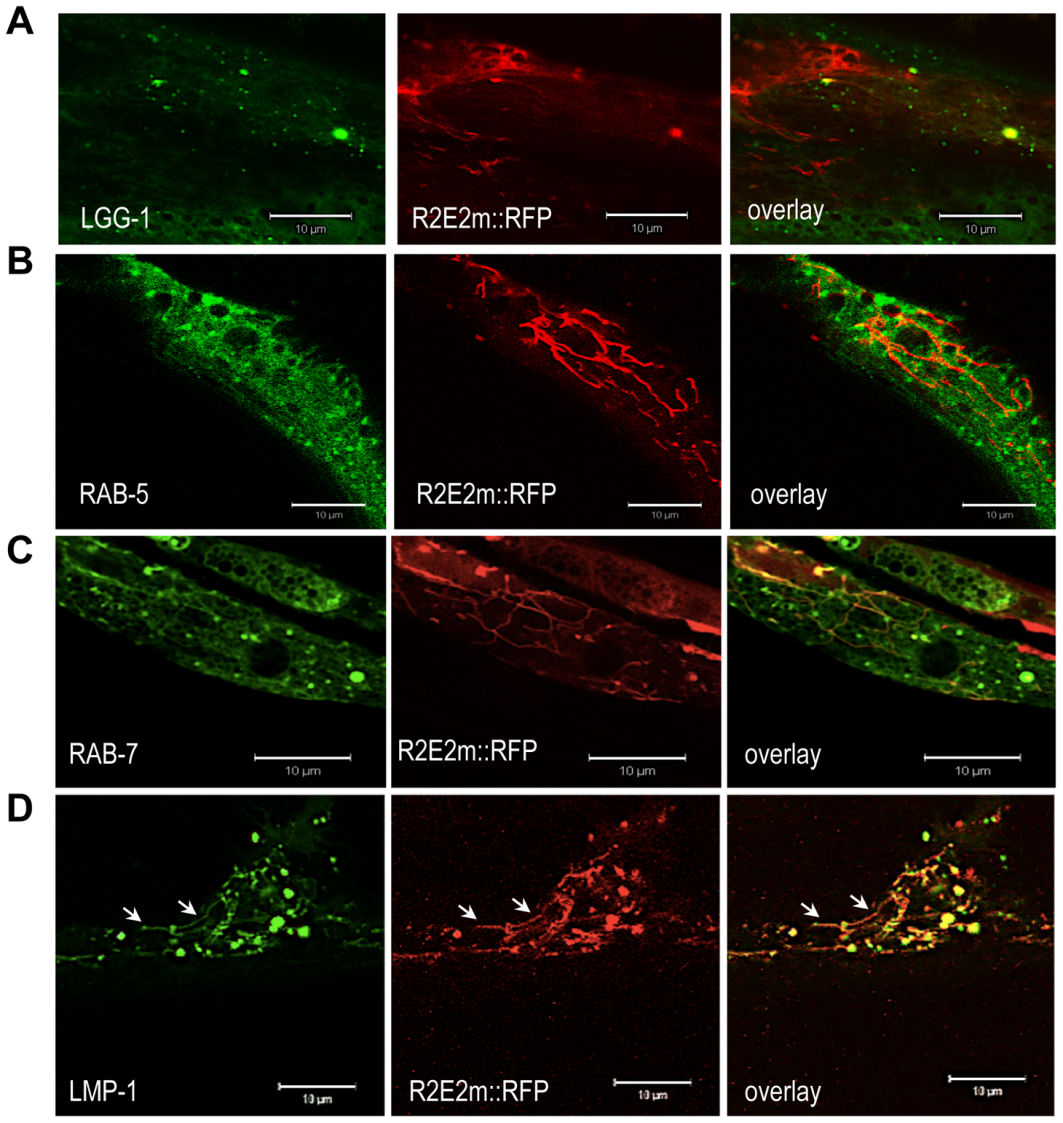
R2E2 co-localizes with autophagosomes, amphisomes, and autolysosomes. (A–D) Confocal images of BWM cells of *C. elegans* expressing R2E2m::RFP together with the indicated vesicle markers. Note that LMP-1 localizes to both tubular shaped vesicles (arrows) and round vesicles. Scale bars: 10 µm.

In R2E2; LGG-1 transgenic lines, we observed co-localization of a subset of R2E2m::RFP foci with autophagosomes (Figure 5A). We also detected co-localization of R2E2m::RFP foci with RAB-7 positive late endosomes and specifically with LMP-1 positive lysosomes (Figure 5C, 5D). The majority of these lysosomes exhibited an unusual tubular shape (Figure 5D). Co-localization was not observed with RAB-5 (early endosomes) (Figure 5B), indicating that the R2E2-containing vesicles were derived from the autophagy pathway rather than from endocytosis. Our studies do not distinguish whether the R2E2m::RFP that co-localizes with vesicular structures corresponds to specific classes of aggregate species described before, as these vesicles have been excluded from FRAP analysis due to their small size (see materials and methods for more details). These data, together with the TEM analysis, suggest that the prion domain is a target of quality-control autophagy and is transferred from autophagosomes to RAB-7 positive amphisomes and LMP-1 positive autolysosomes, respectively.

### Movement of R2E2 between cells and tissues in *C. elegans*

**Figure 6:**
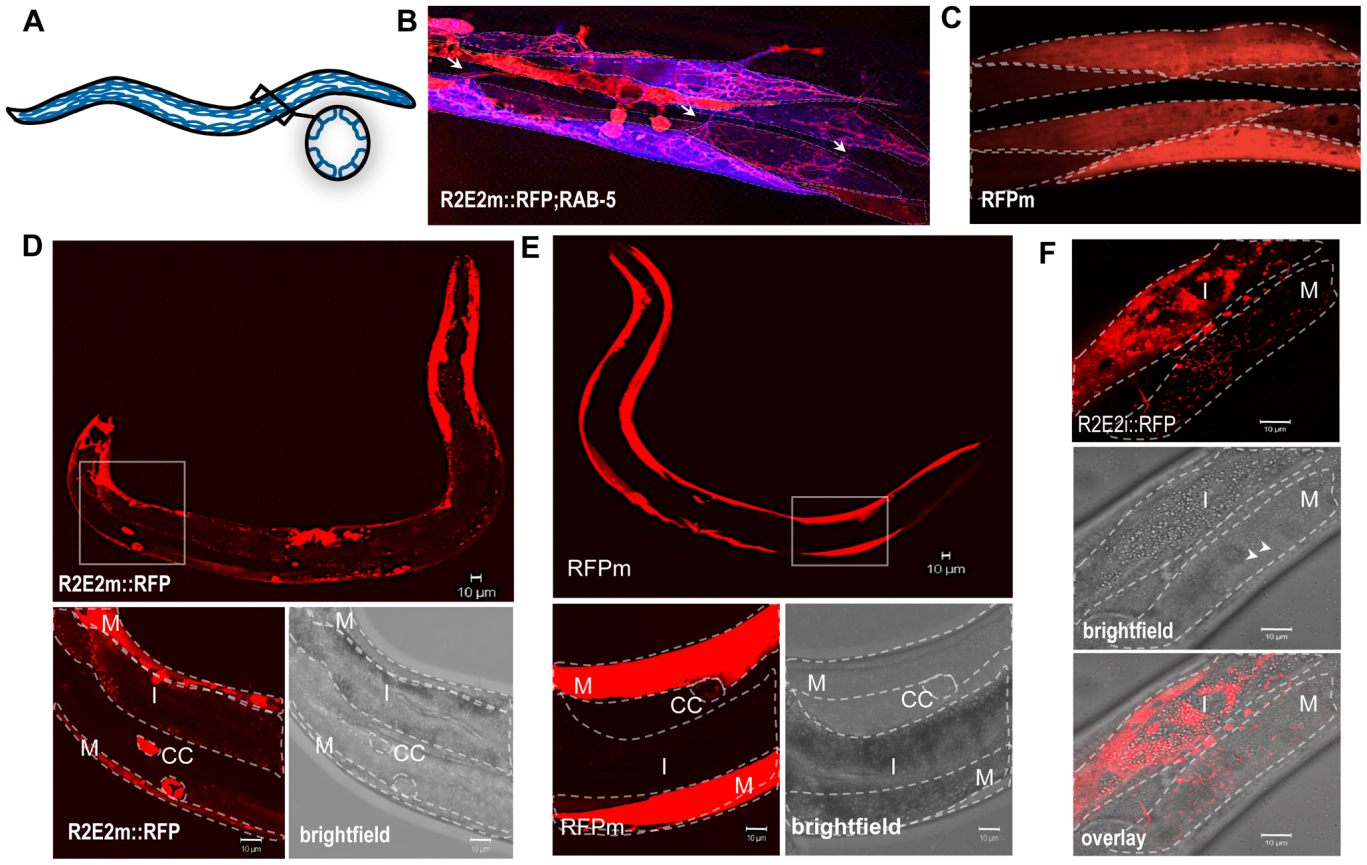
The prion domain spreads between cells and tissues in *C. elegans* by vesicular transport. (A) Nematode muscle organization: BWM cells are arranged in quadrants, four longitudinal bands that run along the entire worm. Each quadrant is formed by staggered pairs of striated mononucleated cells. (B). Collapsed confocal z-stacks of nematodes expressing R2E2m::RFP and CFP::RAB-5 in BWM cells. RAB-5 does not co-localize with R2E2 containing vesicles, but enables the visualization of distinct cells, which is difficult in R2E2m::RFP only expressing nematodes. Single BWM cells of two quadrants are outlined. Arrows indicate connections between cells of different quadrants. Although muscle arms are extended not only to motor neurons but also to distant muscle cells that could form such connections, gap junctions between these cells allow electrical coupling, but no exchange of cytosolic content. (C) Collapsed confocal z-stacks of nematodes expressing RFPm in BWM cells. Single BWM cells of two quadrants are outlined. (D, E) Collapsed confocal z-stack and brightfield images of *C. elegans* expressing R2E2m::RFP or RFPm in BWM cells. Boxed region is displayed in higher magnification. BWM (M), intestine (I) and coelomocytes (CC) are outlined. (F) Single-plane confocal micrographs showing intestinally expressed R2E2i::RFP in vesicles located in intestinal and BWM cells. Intestine (I) and BWM cell (M) are outlined. Note the parallel aligned myofilaments (arrowheads) in the brightfield image as a muscle-specific marker. Scale bars: 10 µm.

Another striking characteristic of the tubular vesicles containing R2E2m::RFP was their dynamic movement within and between muscle cells, monitored by live-cell time-lapse imaging (Video S4, S5; Figure 6B). In particular, the over-expression of RAB-5 enhanced (and facilitated by visualizing single muscle cells) the detection of cell-to-cell transmission of RFP-positive vesicles between BWM cell quadrants (Video S5, Figure 6B). These observations are consistent with previous findings that RAB-5 over-expression increases autophagy [46].

The intercellular transport of R2E2-containing vesicles was unexpected as *C. elegans* body wall muscles are composed of individual mononucleated cells that are connected through gap junctions to allow electrical coupling for coordinated body movement [47] (Figure 6A). No dye coupling has been observed between single muscle cells, implying that there is no unregulated transfer of cytosolic proteins under normal physiological conditions [47]. This leads us to propose that R2E2 is actively transported by tubular vesicles from cell-to-cell. As mentioned before, these vesicles are different from the foci described in Figure 2. Neither the spherical (mobile in FRAP analysis) nor the fibril-like (immobile in FRAP analysis) aggregates are moving within or between cells. Only the small tubular vesicles are getting transmitted and we do not know the conformational state of R2E2 protein within these vesicles. Nevertheless, misfolding and aggregation is central to the toxicity phenotype, as RΔ2-5m::YFP, which does not form these aggregates, exhibits neither a cell autonomous nor cell non-autonomous toxicity.

The moving, tubular-shaped vesicles were only detected in R2E2m::RFP animals, but not observed with the corresponding proteins tagged with YFP. In contrast, the diverse aggregate species and other small vesicular structures (neither tubular nor moving) were visible with both YFP and RFP (compare Figure S1A and Figure 1A, Figure 2B and 2C). In transgenic animals expressing only the RFP fluorescent tag in BWM cells (RFPm), no movement of RFP between cells was observed (Figure 6C, Figure S1B, data not shown). This apparent discrepancy with the fluorescent tags can be explained by RFP being more stable in acidic environments whereas YFP is pH sensitive [48], indicating that these vesicles might exhibit a low lumenal pH that could explain the lack of similar fluorescent structures in R2E2m::YFP expressing animals. This speculation is supported by our results that the moving tubular vesicles co-localize with LMP-1::GFP, but not with LGG-1::GFP (compare Figure 5A and 5D). Indeed, staining of R2E2m::YFP animals with an anti-GFP antibody by indirect immunofluorescence revealed tubular structures in addition to foci visible with YFP fluorescence (Figure S2). This further supports our conclusion that acidified lysosomal vesicles containing the prion domain are transported from cell-to-cell.

Muscle cell-expressed R2E2 was also detected in vesicles of coelomocytes and the intestine (Figure 6D, Figure S3). Both, the intestine and coelomocytes, have been shown to endocytose molecules from the body cavity (pseudocoelom) [49], [50], suggesting that the tubular vesicles containing R2E2 could be released from BWM cells into the pseudocoelom and taken up by endocytosis from surrounding coelomocytes or intestinal cells. While the uptake of proteins from the pseudocoelom into coelomocytes and the intestine is not specific [49], [50], the amount of R2E2m::RFP that accumulates in these tissues is much more pronounced than for RFPm (compare Figure 6D and 6E). These results suggest that R2E2m::RFP is actively released from muscle cells into the pseudocoelom.

To examine the specificity of tissue movement of R2E2, we expressed RFP-tagged R2E2 in intestinal cells and monitored the dynamics of R2E2i::RFP-containing vesicles (Figure S4). Movement of R2E2 was observed by real-time imaging within intestinal cells (Video S6), and from the intestine into adjacent non-expressing muscle cells (Figure 6F, Figure S5, Video S7), thus confirming the spreading of the aggregation-prone prion domain across tissues.

Taken together, these observations reveal that R2E2m::RFP accumulates in tubular vesicles of autolysosomal origin that spread from expressing cells to non-expressing tissues in *C. elegans*. Furthermore, R2E2 seems to spread by two different pathways, either by a direct cell-to-cell transport of lysosomes, or through release into and endocytic uptake from the pseudocoelom (Figure 6B, 6D, 6F, Video S5 and S7).

### R2E2 induces widespread cell non-autonomous protein misfolding

We next examined whether the prion domain induces aggregation of its soluble isoform in *C. elegans*. Such a self-templating or seeding activity forms the basis of amyloid infectivity [9], [22]. To address this, we took advantage of the different aggregation properties of the prion domain constructs and used the non-aggregating RΔ2-5m::YFP as a folding sensor.

**Figure 7:**
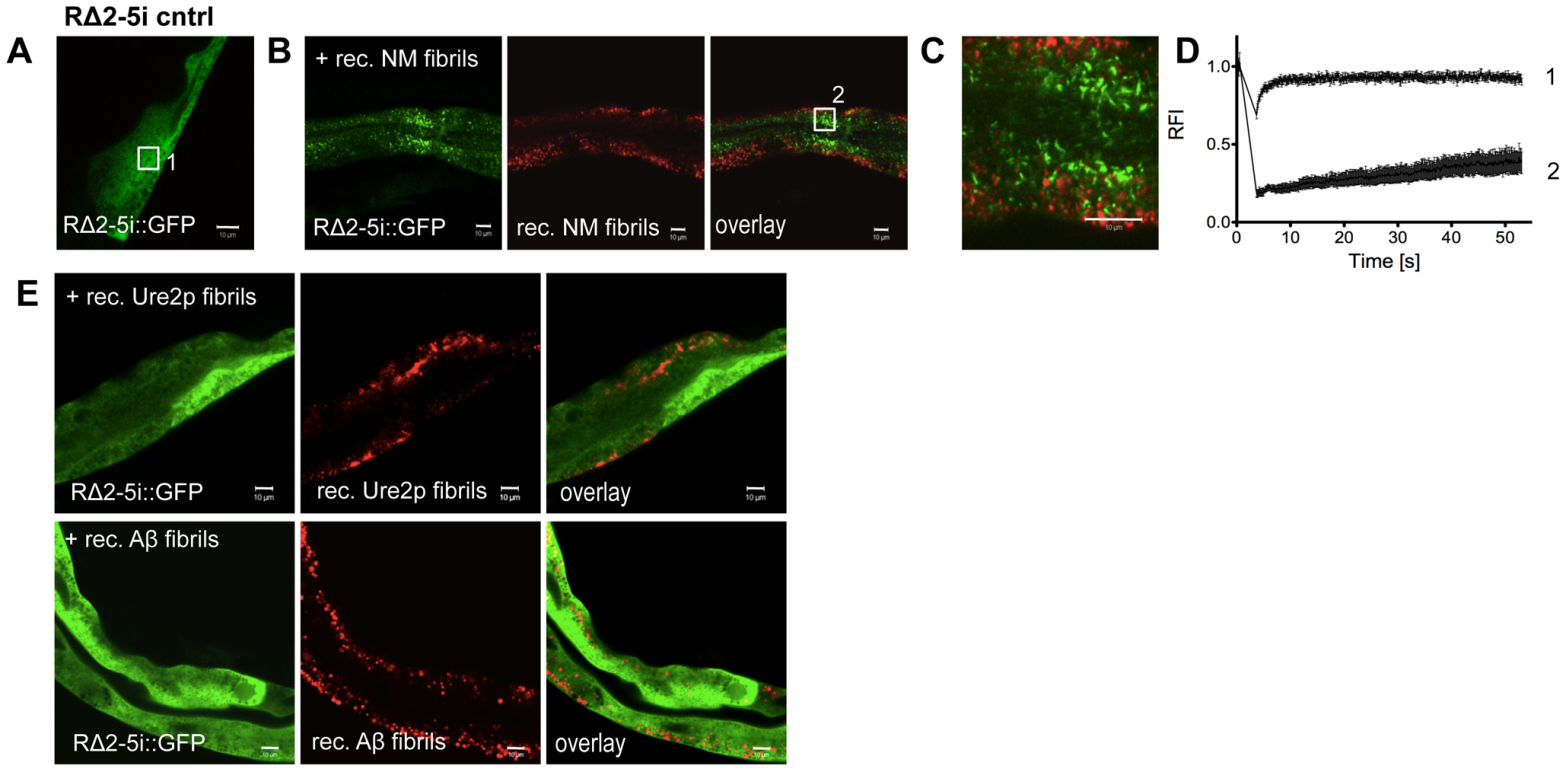
Sequence-specific seeding of RΔ2-5. (A, B) Confocal images of (A) animals expressing GFP-tagged RΔ2-5i::GFP in intestinal cells and (B) 24 h after the injection of Alexa-555-tagged (red) recombinant Sup35NM fibrils. Boxed areas in A and B are representative regions subjected to FRAP analysis. (C) Enlargement of overlay picture in B. (D) FRAP analysis of RΔ2-5i::GFP control (1) and RΔ2-5i::GFP aggregates (2) formed after injection of recombinant Sup35NM fibrils. RFI = relative fluorescence intensity in [%]. (E) Confocal images of animals expressing RΔ2-5i::GFP in the intestine 24 h after injection of the indicated recombinant fibrils.

The seeding model posits a direct interaction of newly forming with preexisting aggregates, which in part is sequence-specific [51]. To examine this, we introduced *in vitro* generated recombinant fibrils by microinjection (Figure S7), into intestinal cells expressing RΔ2-5, as muscle cells were too small for microinjection. These studies were based on previous *in vitro* and cell culture observations that addition of preformed fibrils induces aggregation of the corresponding soluble NM in a sequence-specific manner [9], [44], [52]. We monitored the aggregation state of the RΔ2-5 folding sensor expressed under an intestine-specific (i) promoter (*vha-6p)*. RΔ2-5 and NM constructs exhibited similar patterns of aggregation in the intestine as in muscle cells (Figure S6). Analogous to the biophysical properties exhibited in BWM cells, RΔ2-5i::GFP is soluble in intestinal cells (Figure S6; Figure 7A, 7E). Introduction of recombinant Sup35NM fibrils into intestinal cells resulted in the conversion of RΔ2-5 from a soluble to an aggregated state (Figure 7A, 7B, 7C, 7D) that did not co-localize with the injected Sup35NM fibrils. To address the sequence specificity of these effects, RΔ2-5 animals were also injected with recombinant fibrils of the asparagine-rich yeast prion protein Ure2p with high alpha-helical content [53], [54], or β-sheet rich amyloid Aβ1-42, respectively (Figure 7E). No aggregation of RΔ2-5 was observed upon injection of either protein.

**Figure 8:**
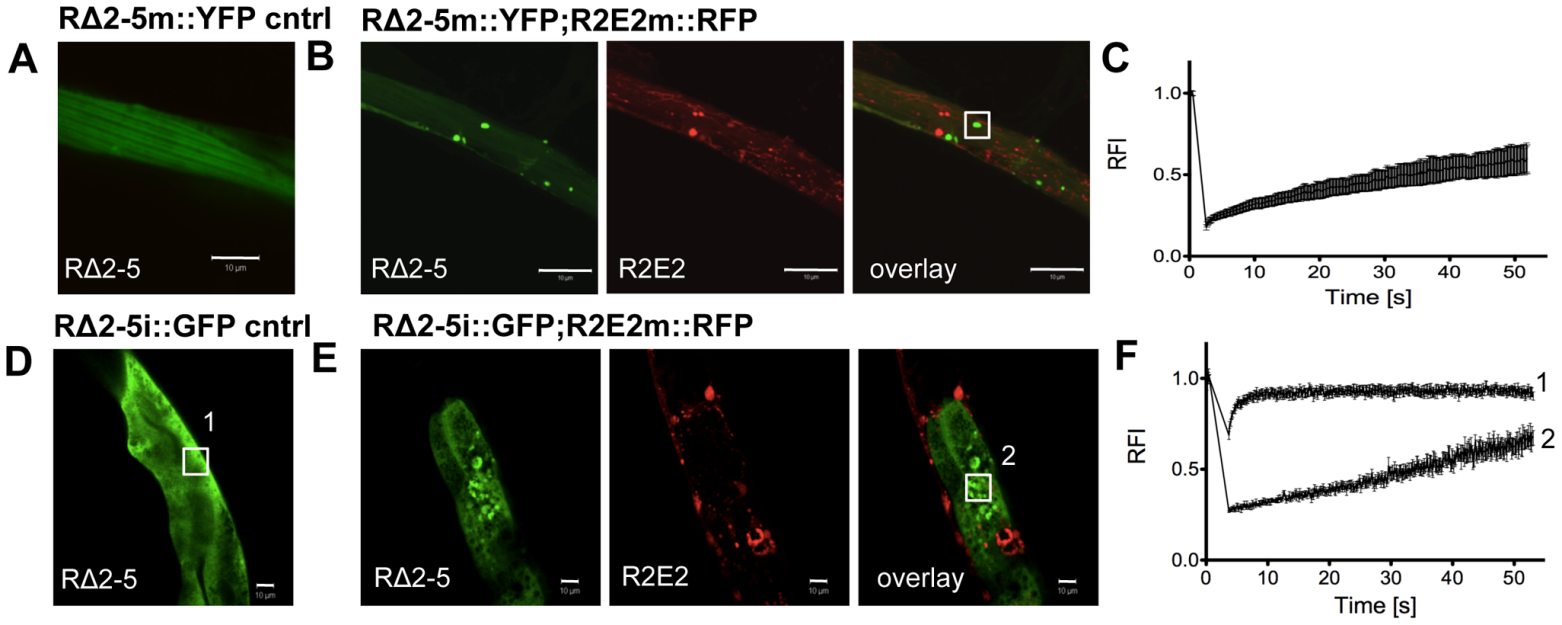
R2E2 induces widespread aggregation of RΔ2-5. (A–C) Co-expression of R2E2m::RFP promotes RΔ2-5m::YFP aggregation. Confocal images of (A) RΔ2-5m::YFP control and (B) RΔ2-5m::YFP co-expressed with R2E2m::RFP in BWM cells. Boxed area indicate representative region used for FRAP analysis. (C) FRAP analysis of RΔ2-5m::YFP foci in a line co-expressing RΔ2-5m::YFP and R2E2m::RFP. (D–F) Muscle-expressed R2E2m::RFP promotes intestinal RΔ2-5i::GFP aggregation. (D) Confocal image of control animal expressing RΔ2-5i::GFP in intestinal cells. (E) Confocal image of animals expressing R2E2m::RFP in BWM cells and RΔ2-5i::GFP in the intestine. Boxed areas indicate representative region analyzed by FRAP. (F) FRAP analysis of RΔ2-5i::GFP alone (1) and in animals co-expressing R2E2m::RFP and RΔ2-5i::GFP (2). RFI = relative fluorescence intensity in [%]. Scale bars: 10 µm.

To test whether cross-seeding occurs when both proteins are co-expressed in *C. elegans* tissues, we crossed RΔ2-5m::YFP with R2E2m::RFP animals. Despite being impaired for spontaneous aggregation, RΔ2-5m::YFP readily formed aggregate species that exhibited slow exchange in FRAP when co-expressed with R2E2m::RFP in BWM cells (Figure 8A, 8B, 8C). RΔ2-5m::YFP aggregates, however, only rarely co-localized with R2E2m::RFP foci (Figure 8B), consistent with observations from the injection experiments (Figure 7B, 7C). The RΔ2-5 sensor was further employed to test whether protein misfolding spreads from R2E2-expressing muscle cells to the intestine. Indeed, aggregation of RΔ2-5i::GFP increased when R2E2m::RFP and RΔ2-5i::GFP were co-expressed (Figure 8E, 8F). The absence of co-localization of RΔ2-5 and R2E2 foci, even when co-expressed (Figure 8B), indicates that aggregation of RΔ2-5 could be due to the global disruption of the folding environment, as seen in tissues co-expressing aggregates of polyglutamine and temperature sensitive mutant proteins [55], rather than from cross-seeding, which would imply co-aggregation of both proteins into heterologous aggregates [51]. Indeed, expression of R2E2 in muscle cells accelerated the age-dependent aggregation of an intestinal polyglutamine (polyQ) folding sensor (Q44i::YFP) [56] (Figure S8).

Taken together, these results show that R2E2m::RFP aggregates have multiple effects by seeding homologous soluble proteins in a sequence-specific manner and causing an imbalance of organismal proteostasis.

## Discussion

**Figure 9:**
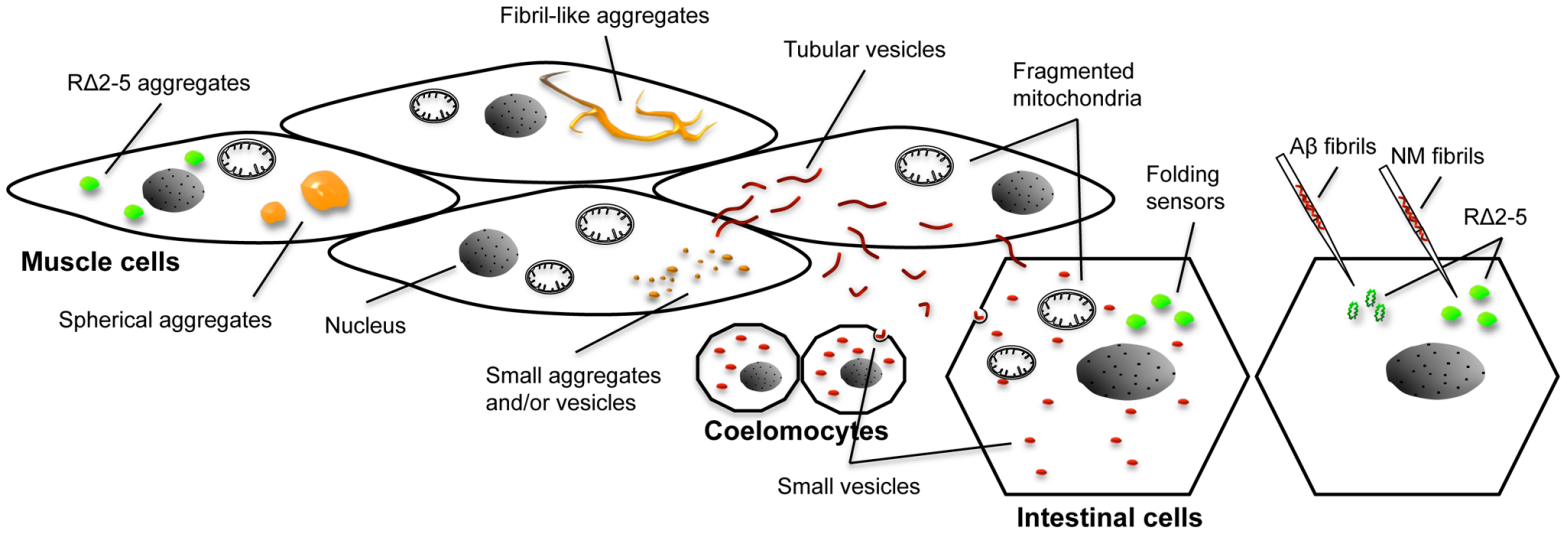
Overview of different aggregate types, vesicular structures, and phenotypes in the *C. elegans* prion model. Expression of R2E2 leads to a range of different foci that were detected with both the YFP and RFP tag (indicated by the orange color). Large foci were analyzed by FRAP and categorized into spherical mobile (containing slowly diffusing protein) and fibrillar immobile aggregates (containing non diffusing protein). Small foci were not assessed by FRAP and consist of presumably both, small aggregates and vesicles (that partially co-localized to LGG-1::GFP). None of the YFP-positive foci exhibited directed movement (very few small foci were moving irregular and undirected within a cell). Fragmented mitochondria were observed in muscle cells and non-expressing tissues by TEM, whereas the YFP signal was not detected outside of body wall muscle cells above the background autofluorescence. In contrast, RFP tagged R2E2 also localized to tubular structures that co-localized with LMP-1::GFP. These tubular structures containing R2E2m::RFP, exhibited directed movement within and between muscle cells (red color indicates vesicles that were only visible with the RFP tag). The RFP-tagged protein was also detected in vesicles of the intestine and coelomocytes, indicating that R2E2 is released from muscle cells and endocytosed by these tissues. Folding sensors are depicted in green. While the injection of recombinant fibrils led to a sequence-specific induction of RΔ2-5 aggregation, co-expression of R2E2 led to a cell autonomous and cell non-autonomous non-sequence specific aggregation of the folding sensors RΔ2-5 and polyQ44. No co-localization of aggregates was observed in both cases.

We have developed a metazoan prion model and examined the properties of a Q/N-rich prion domain in non-dividing terminally differentiated cells using *C. elegans*. A summary model describing the different aggregate species, vesicular structures and phenotypes observed in the *C. elegans* prion model, is shown in Figure 9. As the mechanism of prion propagation differs between unicellular eukaryotes and metazoans, it was unclear whether the prion propensities of Q/N-rich domains are universal and can adapt to different biological systems of cell-to-cell transmission. Spreading of the prion domain from an initial site of expression via autolysosomal vesicles occurs through actively regulated cellular processes, involving a direct transport from cell-to-cell and the release and endocytic uptake of these vesicles from the body cavity. This differs substantially from the propagation of [PSI^+^] in yeast that involves transfer of cytosolic NM propagons from mother to daughter cells during cell divison, that neither requires uptake into membraneous compartments nor active transport. Rather, the transmission of NM between cells and tissues in *C. elegans* is reminiscent of mammalian PrP^Sc^ propagation between post-mitotic neurons. Exosomes [57] and tunneling nanotubes [58] have been suggested as possible routes for cell-to-cell transmission of PrP^Sc^. Either way, cytosolic content also gets transmitted, suggesting that cytosolic and membrane localized prion-like proteins might share some mechanistic aspects of transmission [59]. Indeed, there is now growing evidence that other disease-associated cytosolic protein aggregates can spread from cell-to-cell [19], [20], [26], [34], [35]. The spreading of the prion domain described here in *C. elegans* will allow us to compare the relative potential of tissue transmission with other aggregation-prone amyloidogenic proteins in our model system. It remains to be established if all major disease-associated proteins can spread from cell-to-cell themselves in a similar fashion like NM. Alternatively, prion-like domains might have implications in the spread of pathology throughout the nervous system by allowing a subset of modifiers like FUS and TDP-43 to transmit between cells, which then cause the subsequent aggregation of other disease-linked proteins.

Although motility defects are often associated with the expression of protein aggregates in *C. elegans* muscle cells [38], [39], [41]–[43], the expression of NM was unusually toxic compared to the expression of other disease-associated aggregation-prone proteins [38]–[43]. Aggregation and toxicity of NM were dependent on the oligopeptide repeats. Likewise, in yeast and mammals, the oligorepeats affect spontaneous prion induction and disease prevalence, respectively [44], [60], [61]. In yeast and infected tissue culture cells, prions often elicit no toxicity, suggesting that only non-toxic rapidly replicating variants are selected upon infection in these systems [62]. The *unc-54* promoter used to express NM becomes active post-mitotically in 81 of 95 body wall muscle cells [63], [64]. The toxicity in *C. elegans* could therefore reflect the vulnerability of terminally differentiated non-dividing cells.

Autophagy is important for protein quality control and homeostasis in non-dividing neuronal cells [65], [66], consequently, autophagic failure has been implicated in prion diseases and other neurodegenerative disorders [67]–[69]. While activation of autophagy is beneficial to promote the clearance of disease-associated proteins [70]–[75], the chronic induction of autophagy could have deleterious consequences and may be insufficient to suppress toxicity [76]–[78], in particular if lysosomal function is already compromised during aging or by the chronic expression of mutant proteins [77]–[79]. In line with this, our preliminary results revealed that blocking autophagy by RNAi, to inhibit prion transmission, has only marginal effects to ameliorate NM toxicity in BWM cells (as measured by motility assays, data not shown), indicating that the autophagy-lysosomal pathway has a dual role and also reduces the load of misfolded proteins. Future studies using genome-wide RNAi screens will identify the cellular pathways that improve fitness in these animals.

One of the most striking consequences attributed to expression of the prion domain in *C. elegans* was mitochondrial fragmentation and loss of cristae. An equilibrium of steady fission and fusion events is critical for mitochondrial structure and function, and disruption of this homeostasis has been observed in disease and aging [80]. Intriguingly, a collapse of mitochondrial function was also observed in lysosomal storage disorders associated with impaired lysosomal degradation [81]–[83], and has been proposed to be a common secondary and final mediator of cell death in several diseases associated with autophagic failure and lysosomal dysfunction [81]–[83]. It remains to be determined whether related mechanisms are associated with the disruption of mitochondrial ultrastructure observed here for the *C. elegans* prion model.

There is accumulating evidence that lysosomes have additional roles to their conventional function as digestive organelles. Lysosomes constitute the exosomes of nonsecretory cells [84], are exocytosed during plasma membrane repair [85], and were shown to be transferred via tunneling nanotubes from endothelial progenitor cells to rescue prematurely senescent endothelia [86]. Our results reveal the involvement of lysosomes in the cell-to-cell transmission of cytosolic aggregation-prone proteins. Of note, the exocytosis or transfer of lysosomes may represent a specific cellular response to the prion domain as a cargo, because non mitotic aging tissues fail to secrete indigestible lysosomal content, which leads to the characteristic accumulation of lipofuscin [87]. It is tempting to speculate that proteins with prion domains might trigger a specific cellular response that leads to the release of LMP-1 positive vesicles.

Aggregation of NM in *C. elegans* occurs spontaneously upon its over-expression, in contrast to observations in bacterial and mammalian models [18], [88], [89]. In yeast, the induction of [PSI^+^] is dependent on the co-existence of [PIN^+^] or other compatible aggregation-prone proteins [90]–[92]. Perhaps similar factors such as endogenous Q/N-rich proteins are expressed in *C. elegans* that can act as [PIN^+^] [93].

The injection of preformed fibrils or co-expression of aggregation-prone variants seeds the polymerization of the corresponding monomeric protein [12], [22], [24], [28], [29] by a reaction known as nucleated or seeded polymerization [9], [22]. Only Sup35NM fibrils were able to cross-seed RΔ2-5 to form aggregates, whereas injection of fibrillar Abeta1-42 or Ure2p failed to do so, which suggests that seeding of RΔ2-5 is sequence-specific. However, Sup35NM fibrils or R2E2 aggregates did not co-localize with RΔ2-5 foci. The absence of co-aggregation might be due to conformational variations resulting from sequence differences within the NM oligorepeats [94], [95]. While the different prion domain variants might initially form heterologous seeds below the resolution of our imaging approaches, the preferred coalescence of molecules that have the same conformation might lead to distinct aggregates [51]. Alternatively, the ability to induce polyQ aggregation in a cell non-autonomous manner, suggests that expression of the aggregation-prone prion domain causes a global disruption of cellular proteostasis, and subsequent misfolding of unrelated metastable proteins, perhaps by titrating chaperones and other anti-aggregation factors [55], [90]. Most likely, misfolding of RΔ2-5 upon co-expression of R2E2 in the same or neighboring tissue results from a combinatory effect of sequence-specific cross-seeding together with an overload of the cellular folding capacity. Under these chronic proteotoxic stress conditions, one misfolded protein can accelerate aggregate formation of another aggregation-prone protein independent of protein sequence homology [41], [55].

In summary, this study provides new insights on the intrinsic properties of Q/N-rich prion domains in metazoans. Although the yeast prion domain NM is not a disease relevant peptide, this novel genetic *C. elegans* prion model can elucidate cellular pathways underlying the prion-like propagation of conformational changes in proteins between cells and tissues of multicellular organisms in health and disease.

## Materials and Methods

### Cloning of *C. elegans* expression vectors

Sup35NM constructs were amplified from the yeast expression vector p316CUP1-3SGFP^SG^ [44] containing either the full-length NM, NM with a deletion of oligorepeats # 2-5 (aa 56-93) (RΔ2-5), or NM with a two-times extended oligorepeat # 2 (QGGYQQYNP) (R2E2) [44], by PCR standard methods. Insertion of appropriate restriction sites allowed cloning of the PCR amplicons into pPD30.38 [39]. This vector contains the promoter and enhancer elements from the *unc-54* gene [96], as well as EYFP from the vector pEYFP-N1 (Clontech) [39]. For constructing *myo-3p::sup35(r2e2)::rfp*, *myo-3p::rfp*, *vha-6p::sup35(rΔ2-5)::gfp, vha-6p::sup35(nm)::gfp*, *vha-6p::sup35(r2e5)::rfp, unc-54p::cfp::rab-5*, and *unc-54p::lmp-1::gfp* expression vectors, the MultiSite Gateway Three-Fragment Vector Construction Kit (Invitrogen) was used. NM constructs were amplified from the pPD30.38 vectors using appropriate oligonucleotides for gateway cloning and inserted into the pDONR 221 entry vector by recombination. Likewise, the *lmp-1* coding sequence was amplified from a N2 cDNA sample and inserted into the pDONR 221 entry vector. The plasmid pCZGY#3 ( = pDONR 201_*rab-5*) was a kind gift from Dr. Yishi Jin. Entry vectors pDONR P4-P1R containing *myo-3* (approx. 2.4 kb upstream of the *myo-3* gene), *vha-6* (approx. 1.2 kb upstream of the *vha-6* gene), or *unc-54* (approx. 1 kb upstream of the *unc-54* gene) promoter region and pDONR P2R-P3 coding for the C-terminal monomeric RFP or GFP tag, were generated accordingly. The N-terminal CFP was cloned between the *unc-54* promoter and *rab-5* using appropriate restriction sites. For co-localization, CFP::Rab-5 was false-colored green. All pDONR P2R-P3 entry vectors also contained the *unc-54* 3′UTR. Promoters, genes of interest and fluorescent tags were finally inserted into the destination vector pDEST R4-R3 in an *in vitro* recombination reaction.

### *C. elegans* methods

Wild-type (N2, Bristol) and transgenic worms were handled using standard methods [97]. If not otherwise indicated, nematodes were grown on NGM plates seeded with the *E. coli* strain OP50 at 20°C. The strains NP1129 *cdIs131[cc1p::gfp::rab-5+unc-119(+)+myo-2::gfp]*, NP871 *cdIs66[cc1p::gfp::rab-7+unc-119(+)+myo-2::gfp]*, NP744 *cdIs39[cc1p::gfp::rme-1(271alpha1)+unc-119(+)+myo-2::gfp]*, RT258 *pwIs50[lmp-1p::lmp-1::gfp+Cb-unc-119(+)]*, and DA2123 *adIs2122[lgg-1::GFP + rol-6(su1006)]* were ordered from the Caenorhabditis Gene Center (CGC). The strain FY777 *lin-15(n765ts); grEx170[Pmyo-3::gfp::rab-7; lin-15(+)]* was a kind gift of Dr. Bruce Bamber. The following strains were generated for this study using germline transformation by microinjection:

AM801 *rmIs319[unc-54p::sup35(rΔ2-5)::yfp]*, AM803 *rmIs321[unc-54p::sup35(nm)::yfp]*, AM806 *rmIs324[unc-54p::sup35(r2e2)::yfp]*, AM815 *rmIs323[myo-3p::sup35(r2e2)::rfp]*, AM809 *rmEx319[vha-6p::sup35(rΔ2-5)::gfp+myo-2p::mcherry]*, AM823 *rmEx326[vha-6p::sup35(rΔ2-5)::gfp], AMf814 rmIs326[vha-6p::sup35(nm)::gfp+myo-2p::mcherry], AM883 rmEx338[myo-3p::rfp::rfp], AM887 rmEx339[unc-54p::cfp::rab-5], AM890 rmEx340[unc-54p::lmp-1::gfp]*.

Transgenic lines carrying extrachromosomal arrays were generated by microinjection of the above-mentioned plasmids into N2 wild-type animals. Integrations were obtained by gamma irradiation of animals carrying the respective extrachromosomal array. Integrated lines were backcrossed at least five times. Importantly, due to the high toxicity of some of the transgenes, the lines had to be backcrossed into N2 wild-type background regularly to avoid the occurrence of mutations that improve the health or suppress the NM aggregation phenotype of the transgenic lines. For the same reason, assays were performed on freshly backcrossed or crossed animals.

### Developmental assays

Nematodes were synchronized by transferring adult animals on a fresh plate and were allowed to lay eggs for 2.5 hours before removing. The amount of eggs laid was determined. Embryos were grown at 20°C for 72 hours before assessment of their developmental stage. Data obtained in at least three independent experiments were analyzed (≥200 worms total). In parallel, 20 L1 larvae were picked on fresh plates and grown for four days at 20°C before pictures and movies were taken.

### Motility assay

L4 larvae from N2 and transgenic lines were transferred on fresh plates. Movement of crawling animals was recorded 24 hours later (with young adult worms) using a Leica M205 FA microscope with a Hamamatsu digital camera C10600-10B (Orca-R2, Leica Microsystems, Switzerland), and the Hamamatsu Simple PCI Imaging software. Videos were imported into ImageJ and speed (measured as body length per second) was analyzed using the wrMTrck plugin for ImageJ. Each sample containing 20–30 worms was recorded three times and the average speed of these movies was considered one biological sample. At least three biological replicates were obtained for each strain tested.

### Immunofluorescence

For rhodamine-phalloidin staining, transgenic lines were fixed (4% formaldehyde solution), permeabilized (130 mM Tris, pH 6.8; 700 mM ß-mercaptoethanol; 1% Triton X-100) and stained with rhodamine-phalloidin (Molecular Probes). For indirect antibody staining of R2E2, R2E2m::YFP animals were washed in M9, transferred onto Poly-L-Lysine coated microscope slides (Electron Microscopy Sciences), covered with coverslips and frozen on a metal block chilled to about −70°C on dry ice. The coverslips were snapped off and the slides were fixed in −20°C methanol, washed twice (1x PBS), blocked (1x PBS; 4% BSA; 0.1% Triton X-100), and incubated with anti-GFP antibody (ab6556 from Abcan) in blocking solution at 4°C over night. The next day, slides were washed 4x (1x PBS), incubated with secondary antibody (Alexa-456 conjugated goat anti-rabbit IgG, Invitrogen) for 1 h at room temperature, before being washed again, mounted (PermaFluor Aqueous Mounting Medium, Thermo Scientific) and sealed.

### Imaging and FRAP

For light and fluorescence microscopy, animals were mounted on 2% agarose pads and immobilized in 2 mM levamisole. DIC (Nomarski) images were obtained using a Zeiss Axiovert 200 microscope with a Hamamatsu Orca 100 cooled CCD camera. Fluorescence microscopy and FRAP analysis were performed on a Zeiss LSM 510 confocal microscope with a 488 nm, 514 nm, or 563 nm line for excitation.

FRAP was performed by using the 63 x objective lens at 5 x zoom, with the 514 or 488 nm line for excitation of YFP or GFP, respectively. An area of 0.623 µm^2^ was bleached for 50 iterations at 100% transmission, after which time an image was collected every 123.35 ms. The relative fluorescence intensity (RFI) was determined by using RFI = (T_t_/C_t_)/(T_0_/C_0_), where T_0_ denotes the fluorescence intensity of the bleached region and C_0_ the control unbleached region, prior to bleaching, and T_t_ and C_t_ represent the fluorescence intensity at time t after photobleaching for the bleached and control region, respectively. Results show an average of at least 20 independent measurements for each strain. Foci that got rapidly and evenly bleached allover beyond the outline of the bleached region of interest (ROI) were excluded from these analysis, as they likely constitute vesicles containing soluble protein. In addition, foci, that had the same or a smaller size than the bleached ROI of 0.623 µm^2^ were not taken into account, as the motility of the protein within the same focus could not be assessed and therefore, vesicles containing soluble protein could not be distinguished from aggregates. FRAP analysis of RΔ2-5 foci in RΔ2-5m::YFP;R2E2m::RFP animals, RΔ2-5i::GFP animals 24 h after injected with rec. fibrils, and RΔ2-5i::GFP;R2E2m::RFP animals were performed on young adult (day 1 and 2 of adulthood) worms. Where indicated, a Leica SP5 II LSCM equipped with HyD detectors was used, especially for time-lapse imaging.

### Worm lysis and detergent solubility assay

Nematodes were collected from a densely populated not starved 6 cm or 10 cm plate, washed in M9 buffer, and resuspended in lysis buffer (20 mM Tris, pH 7.5; 10 mM ß-mercaptoethanol; 0.5% Triton X-100; supplemented with complete protease inhibitor (Roche)) before shock freezing in liquid nitrogen. After three freeze-thaw cycles, the worm pellet was grinded with a motorized pestle, and lysed on ice, in the presence of 0.025 U/ml benzonase (Sigma). The lysate was centrifuged at 1000 rpm for 1 min in a tabletop centrifuge to pellet the carcasses. Protein concentration was determined using Bradford assay (Bio-Rad).

For the solubility assay, 200 µg of total protein was mixed with 2% N-Lauroylsarcosine, before ultracentrifugation at 100.000 g for 1 hour at 4°C. Supernatant and pellet fractions were separated and subjected to SDS-PAGE and subsequent immunoblotting. For transgene detection, the mouse anti-GFP monoclonal antibody (MMS-118R, Covance) was used, together with ECL plus (GE Healthcare). As a loading control, α-tubulin was detected by anti-α-tubulin antibody (T-5168, Sigma).

### Procedures for *C. elegans* Transmission Electron Microscopy (TEM) analysis

Samples were high pressure frozen (HPF) using a Leica EM PACT2, and maintained in LN2 until processed at low temperature in an automated freeze substitution unit (Leica EM AFS2). The freeze substitution solution (2% OsO_4_, 0.5% uranyl acetat, 3% H_2_0 in Acetone) was cooled to −90°C before adding the HPF sample. Low temperature processing was performed over 3 days where the temperature was gradually increased to room temperature, followed by a gradual infiltration with EMBed 812 resin and polymerization. 90 nm thin sections were collected on Formvar-coated slot grids and stained with 2% uranyl acetate and Reynold's lead citrate. Samples were imaged at 80 kV in a JEOL 1230 transmission electron microscope. 4 different R2E2 expressing animals and 2 N2 animals were imaged.

### Aggregate quantification

Animals were synchronized via bleaching as described earlier [39]. Synchronized L1 larvae were transferred on fresh OP50-seeded plates ( = day 1). Animals were observed at 30–40x magnification with a stereomicroscope equipped for epifluorescence. The number of animals containing intestinal polyQ aggregates was determined on day 1 to 5 after synchronization. At least 300 animals (total) were assessed in 3 independent experiments.

### Recombinant fibril preparation and labeling

Sup35NM, Aß (1–42), and full-length Ure2p expression, purification and assembly were performed as described [98]–[100]. Sup35NM, Aß (1–42) and Ure2p fibrils were spun at 15.000 g for 15 min at 4°C. The fibrils were resuspended in 50 mM HEPES, pH 7.5. Labeling was achieved by addition of 2 molar excess of the aminoreactive fluorescent dye Alexa Fluor 555 carboxylic acid, succinimidyl ester (Invitrogen) following the manufacturer's recommendations. Unreacted dye was removed by 3 cycles of sedimentation and resuspension of the fibrils in HEPES buffer. The amount and quality of recombinant fibrils was determined by solubility assay (16.000 g, 30 min, 4°C) and TEM.

### TEM analysis of recombinant fibrils

The fibrillar nature of the generated assemblies was assessed using a JEOL 1400 transmission electron microscope following adsorption of the samples onto carbon-coated 200-mesh grids and negative staining with 1% uranyl acetate. The images were recorded with a Gatan Orius CCD camera.

### Injection of recombinant protein

Immediately before injection, recombinant fibrils were diluted into 50 mM HEPES buffer, pH 7.5, to a final concentration of 100 µM and sonicated (1510 Branson water sonicator) for 30 min. 50 mM HEPES buffer only was used as a control. Young adult worms were microinjected according to standard methods into the cytosol of intestinal cells. After 24 h nematodes were imaged using a Zeiss LSM 510 confocal microscope. At least 5 animals were injected with each fibril preparation and analyzed per experiment and experiments were repeated 2–3 times. Our microinjection setup does not allow controlling for the injection of the exact same amount of protein fibrils into each animal. Therefore, we assessed different concentrations of the fibril preparations in their ability to induce RΔ2-5 aggregation. The fibril concentration had only an impact on the quantity (how much protein was seeded), but not on the quality (if there was seeding) of aggregation induction.

## Supporting Information

Figure S1 Detailed description of aggregates and vesicular structures occuring in R2E2m::RFP and RFPm expressing animals. (A) Collapsed confocal z-stack images of R2E2m::RFP expressing nematodes reveal tubular vesicles in addition to multiple types of aggregate structures. (B) The RFP tag alone remains mostly soluble upon expression in BWM cells, but was also occasionally found in vesicular structures and small aggregates. The majority of these vesicles remained stationary (data not shown), indicating that although RFP is able to visualize acidic vesicles, the occurrence of moving tubular vesicles is associated with the aggregation-prone prion domain. Open arrow and diamond arrow point to large round, and fibril-like aggregates, respectively. Arrowheads indicate small aggregates (indistinguishable from small round vesicles) or round and tubular vesicles.
(TIF)

Figure S2 R2E2m::YFP does form vesicular structures that can be visualized by indirect immunofluorescence. (A) R2E2m::YFP stained with anti-GFP antibody. The GFP staining reveals vesicular structures that are not visible with YFP fluorescence. The fibril-like aggregate was overexposed in both channels (YFP and RFP) to better show vesicular structures in RFP and to demonstrate the absence of YFP-positive vesicular structures. (B) N2 wild-type control stained with anti-GFP antibody. No unspecific staining of the anti-GFP antibody was observed. Scale bars: 10 µm.
(TIF)

Figure S3 R2E2m::RFP gets released into the body cavity and endocytosed by coelomocytes. Confocal fluorescence and brightfield images of animals carrying the indicated coelomocyte vesicle markers and expressing R2E2m::RFP in BWM cells. Coelomocytes are outlined. R2E2m::RFP does co-localize with (A) early (RAB-5) and (B) late (RAB-7) endosomes, and (C) lysosomes (LMP-1), but not with (D) recycling endosomes (RME-1). Scale bars: 10 µm.
(TIF)

Figure S4 R2E2i::RFP containing vesicles emerge also in *C. elegans* intestinal cells. Confocal fluorescence and brightfield images of animals expressing R2E2i::RFP in the intestine. Scale bar: 10 µm.
(TIF)

Figure S5 R2E2i::RFP containing vesicles are able to exit from *C. elegans* intestinal cells and spread into non-expressing tissues. Confocal fluorescence and brightfield images of animals expressing R2E2i::RFP in intestinal cells. Intestine (I) and coelomocytes (CC) are outlined. Arrows indicate distant vesicles that have been release from the intestine. As the low fluorescence of released vesicles reaches the detection limit of the microscope, the RFP channel was overexposed to better show vesicular structures outside of the intestine. Scale bar: 10 µm.
(TIF)

Figure S6 Oligopeptide repeat region dependent aggregation of the prion domain in intestinal cells. Collapsed confocal z-stack images of *C. elegans* lines stably expressing the indicated transgene. Pictures were taken at displayed stages during nematode development (embryo/L2 larvae/adult). Scale bar: 10 µm.
(TIF)

Figure S7 Quality control of fibrilized proteins used for microinjection. SDS solubility assays (A) and TEM images (B) of indicated fibrilized proteins. P = pellet, S = supernatant. Scale bars: 0.1 µm in the panels Sup35NM and Ure2p, 0.01 µm in the panel Aβ.
(TIF)

Figure S8 R2E2m::RFP induces cell non-autonomous aggregation of Q44i::YFP. (A). Representative fluorescent images of Q44i::YFP and Q44i::YFP;R2E2m::RFP animals on day 4 after transferring synchronized L1 larvae onto fresh OP50-seeded NGM plates. The expression of R2E2 in BWM cells led to an earlier onset of Q44 aggregation in the intestine. (B). Quantification of animals with aggregates (in %) on indicated days after synchronization. Error bars: S.D.
(TIF)

Video S1 20 L1 larvae of RΔ2-5m::YFP expressing animals were grown for four days before movie was taken over a time of 30 s with a speed of 2.8 frames per second (fps). Video is played at 5 fps. No reduction in motility or developmental delay was observed.
(AVI)

Video S2 20 L1 larvae of NMm::YFP expressing *C. elegans* were grown for four days before movie was taken over a time of 30 s with a speed of 2 fps. Video is played at 5 fps. Not only reduced motility, but also developmental delay and less progeny was observed in comparison to RΔ2-5 animals.
(AVI)

Video S3 20 L1 larvae of R2E2m::YFP transgenic lines were grown for four days before movie was taken over a time of 30 s with a speed of 2.8 fps. Video is played at 5 fps. R2E2 animals were frequently paralyzed and exhibited severe developmental defects and sterility. Note the significantly reduced number of tracks on the bacterial lawn, indicating less total number of animals in comparison to NM animals.
(AVI)

Video S4 A fraction of R2E2m::RFP containing vesicles is moving within and between *C. elegans* BWM cells. Nematodes expressing R2E2m::RFP were mounted and movie of z-stacks (xyzt) was taken using a Leica SP5 confocal microscope. This movie shows 53 stacks of a 3 µm thick section (with 7 frames taken every 0.5 µm). One stack was taken every 5.3 seconds. Video is played at 12 stacks per seconds. See also Figure S1AII, a representative picture of this movie.
(AVI)

Video S5 R2E2m::RFP containing vesicles are able to transmit between *C. elegans* BWM cells. Nematodes expressing R2E2m::RFP and RAB-5::CFP in BWM cells were mounted and movie was taken using a Leica SP5 confocal microscope. 180 frames (90 frames per channel) were taken with a speed of 0.74 fps. Video is played at 12 fps. Scale bar: 25 µm. See also Figure 6B.
(AVI)

Video S6 A fraction of R2E2i::RFP containing vesicles is moving within *C. elegans* intestinal cells. Nematodes expressing R2E2m::RFP were mounted and movies were taken using a Zeiss LSM510 confocal microscope. 99 frames were taken with a speed of 0.5 fps. Video is played at 20 fps. See also Figure S4, a representative picture of this movie.
(AVI)

Video S7 R2E2i::RFP containing vesicles are able to move from *C. elegans* intestinal cells into non-expressing tissues. Nematodes expressing R2E2i::RFP were mounted and movies were taken using a Zeiss LSM510 confocal microscope. 161 frames were taken with a speed of 0.5 fps. Video is played at 20 fps. As the low fluorescence of released vesicles reaches the detection limit of the microscope, the RFP channel was overexposed to better show vesicular structures outside of the intestine, which results in a low video quality. Figure S5 shows a representative picture of this movie and outlines the tissues and highlights the released vesicles.
(AVI)

## References

[1] Prusiner SB (1982) Novel proteinaceous infectious particles cause scrapie. Science 216: 136–144. 6801762 10.1126/science.6801762

[2] Jarrett JT, Lansbury PT Jr (1993) Seeding “one-dimensional crystallization” of amyloid: a pathogenic mechanism in Alzheimer's disease and scrapie? Cell 73: 1055–1058. 8513491 10.1016/0092-8674(93)90635-4

[3] Pan KM, Baldwin M, Nguyen J, Gasset M, Serban A, et al (1993) Conversion of alpha-helices into beta-sheets features in the formation of the scrapie prion proteins. Proc Natl Acad Sci U S A 90: 10962–10966. 7902575 10.1073/pnas.90.23.10962 PMC47901

[4] Sailer A, Bueler H, Fischer M, Aguzzi A, Weissmann C (1994) No propagation of prions in mice devoid of PrP. Cell 77: 967–968. 7912659 10.1016/0092-8674(94)90436-7

[5] Caughey B, Kocisko DA, Raymond GJ, Lansbury PT Jr (1995) Aggregates of scrapie-associated prion protein induce the cell-free conversion of protease-sensitive prion protein to the protease-resistant state. Chem Biol 2: 807–817. 8807814 10.1016/1074-5521(95)90087-x

[6] Beekes M, McBride PA, Baldauf E (1998) Cerebral targeting indicates vagal spread of infection in hamsters fed with scrapie. J Gen Virol 79 Pt 3: 601–607. 9519840 10.1099/0022-1317-79-3-601

[7] Kimberlin RH, Walker CA (1982) Pathogenesis of mouse scrapie: patterns of agent replication in different parts of the CNS following intraperitoneal infection. J R Soc Med 75: 618–624. 6809940 10.1177/014107688207500809 PMC1438061

[8] Wickner RB (1994) [URE3] as an altered URE2 protein: evidence for a prion analog in Saccharomyces cerevisiae. Science 264: 566–569. 7909170 10.1126/science.7909170

[9] Glover JR, Kowal AS, Schirmer EC, Patino MM, Liu JJ, et al (1997) Self-seeded fibers formed by Sup35, the protein determinant of [PSI+], a heritable prion-like factor of S. cerevisiae. Cell 89: 811–819. 9182769 10.1016/s0092-8674(00)80264-0

[10] Serio TR, Lindquist SL (1999) [PSI+]: an epigenetic modulator of translation termination efficiency. Annu Rev Cell Dev Biol 15: 661–703. 10611975 10.1146/annurev.cellbio.15.1.661

[11] Serio TR, Cashikar AG, Kowal AS, Sawicki GJ, Moslehi JJ, et al (2000) Nucleated conformational conversion and the replication of conformational information by a prion determinant. Science 289: 1317–1321. 10958771 10.1126/science.289.5483.1317

[12] Tanaka M, Chien P, Naber N, Cooke R, Weissman JS (2004) Conformational variations in an infectious protein determine prion strain differences. Nature 428: 323–328. 15029196 10.1038/nature02392

[13] Chernoff YO, Lindquist SL, Ono B, Inge-Vechtomov SG, Liebman SW (1995) Role of the chaperone protein Hsp104 in propagation of the yeast prion-like factor [psi+]. Science 268: 880–884. 7754373 10.1126/science.7754373

[14] Ter-Avanesyan MD, Kushnirov VV, Dagkesamanskaya AR, Didichenko SA, Chernoff YO, et al (1993) Deletion analysis of the SUP35 gene of the yeast Saccharomyces cerevisiae reveals two non-overlapping functional regions in the encoded protein. Mol Microbiol 7: 683–692. 8469113 10.1111/j.1365-2958.1993.tb01159.x

[15] Neumann M, Kwong LK, Truax AC, Vanmassenhove B, Kretzschmar HA, et al (2007) TDP-43-positive white matter pathology in frontotemporal lobar degeneration with ubiquitin-positive inclusions. J Neuropathol Exp Neurol 66: 177–183. 17356379 10.1097/01.jnen.0000248554.45456.58

[16] Kwiatkowski TJ Jr, Bosco DA, Leclerc AL, Tamrazian E, Vanderburg CR, et al (2009) Mutations in the FUS/TLS gene on chromosome 16 cause familial amyotrophic lateral sclerosis. Science 323: 1205–1208. 19251627 10.1126/science.1166066

[17] Cushman M, Johnson BS, King OD, Gitler AD, Shorter J (2010) Prion-like disorders: blurring the divide between transmissibility and infectivity. J Cell Sci 123: 1191–1201. 20356930 10.1242/jcs.051672 PMC2848109

[18] Krammer C, Schatzl HM, Vorberg I (2009) Prion-like propagation of cytosolic protein aggregates: insights from cell culture models. Prion 3: 206–212. 19901539 10.4161/pri.3.4.10013 PMC2807693

[19] Aguzzi A (2009) Cell biology: Beyond the prion principle. Nature 459: 924–925. 19536253 10.1038/459924a

[20] Soto C (2012) Transmissible proteins: expanding the prion heresy. Cell 149: 968–977. 22632966 10.1016/j.cell.2012.05.007 PMC3367461

[21] Jarrett JT, Lansbury PT Jr (1992) Amyloid fibril formation requires a chemically discriminating nucleation event: studies of an amyloidogenic sequence from the bacterial protein OsmB. Biochemistry 31: 12345–12352. 1463722 10.1021/bi00164a008

[22] Harper JD, Lansbury PT Jr (1997) Models of amyloid seeding in Alzheimer's disease and scrapie: mechanistic truths and physiological consequences of the time-dependent solubility of amyloid proteins. Annu Rev Biochem 66: 385–407. 9242912 10.1146/annurev.biochem.66.1.385

[23] Scherzinger E, Sittler A, Schweiger K, Heiser V, Lurz R, et al (1999) Self-assembly of polyglutamine-containing huntingtin fragments into amyloid-like fibrils: implications for Huntington's disease pathology. Proc Natl Acad Sci U S A 96: 4604–4609. 10200309 10.1073/pnas.96.8.4604 PMC16379

[24] Busch A, Engemann S, Lurz R, Okazawa H, Lehrach H, et al (2003) Mutant huntingtin promotes the fibrillogenesis of wild-type huntingtin: a potential mechanism for loss of huntingtin function in Huntington's disease. J Biol Chem 278: 41452–41461. 12888569 10.1074/jbc.M303354200

[25] Krebs MR, Morozova-Roche LA, Daniel K, Robinson CV, Dobson CM (2004) Observation of sequence specificity in the seeding of protein amyloid fibrils. Protein Sci 13: 1933–1938. 15215533 10.1110/ps.04707004 PMC2279934

[26] Meyer-Luehmann M, Coomaraswamy J, Bolmont T, Kaeser S, Schaefer C, et al (2006) Exogenous induction of cerebral beta-amyloidogenesis is governed by agent and host. Science 313: 1781–1784. 16990547 10.1126/science.1131864

[27] Frost B, Jacks RL, Diamond MI (2009) Propagation of tau misfolding from the outside to the inside of a cell. J Biol Chem 284: 12845–12852. 19282288 10.1074/jbc.M808759200 PMC2676015

[28] Danzer KM, Krebs SK, Wolff M, Birk G, Hengerer B (2009) Seeding induced by alpha-synuclein oligomers provides evidence for spreading of alpha-synuclein pathology. J Neurochem 111: 192–203. 19686384 10.1111/j.1471-4159.2009.06324.x

[29] Ren PH, Lauckner JE, Kachirskaia I, Heuser JE, Melki R, et al (2009) Cytoplasmic penetration and persistent infection of mammalian cells by polyglutamine aggregates. Nat Cell Biol 11: 219–225. 19151706 10.1038/ncb1830 PMC2757079

[30] Kane MD, Lipinski WJ, Callahan MJ, Bian F, Durham RA, et al (2000) Evidence for seeding of beta -amyloid by intracerebral infusion of Alzheimer brain extracts in beta -amyloid precursor protein-transgenic mice. J Neurosci 20: 3606–3611. 10804202 10.1523/JNEUROSCI.20-10-03606.2000 PMC6772682

[31] Munch C, O'Brien J, Bertolotti A (2011) Prion-like propagation of mutant superoxide dismutase-1 misfolding in neuronal cells. Proc Natl Acad Sci U S A 108: 3548–3553. 21321227 10.1073/pnas.1017275108 PMC3048161

[32] Lundmark K, Westermark GT, Nystrom S, Murphy CL, Solomon A, et al (2002) Transmissibility of systemic amyloidosis by a prion-like mechanism. Proc Natl Acad Sci U S A 99: 6979–6984. 12011456 10.1073/pnas.092205999 PMC124514

[33] Eisele YS, Obermuller U, Heilbronner G, Baumann F, Kaeser SA, et al (2010) Peripherally applied Abeta-containing inoculates induce cerebral beta-amyloidosis. Science 330: 980–982. 20966215 10.1126/science.1194516 PMC3233904

[34] Li JY, Englund E, Holton JL, Soulet D, Hagell P, et al (2008) Lewy bodies in grafted neurons in subjects with Parkinson's disease suggest host-to-graft disease propagation. Nat Med 14: 501–503. 18391963 10.1038/nm1746

[35] Clavaguera F, Bolmont T, Crowther RA, Abramowski D, Frank S, et al (2009) Transmission and spreading of tauopathy in transgenic mouse brain. Nat Cell Biol 11: 909–913. 19503072 10.1038/ncb1901 PMC2726961

[36] de Calignon A, Polydoro M, Suarez-Calvet M, William C, Adamowicz DH, et al (2012) Propagation of tau pathology in a model of early Alzheimer's disease. Neuron 73: 685–697. 22365544 10.1016/j.neuron.2011.11.033 PMC3292759

[37] Luk KC, Kehm VM, Zhang B, O'Brien P, Trojanowski JQ, et al (2012) Intracerebral inoculation of pathological alpha-synuclein initiates a rapidly progressive neurodegenerative alpha-synucleinopathy in mice. J Exp Med 209: 975–986. 22508839 10.1084/jem.20112457 PMC3348112

[38] Link CD (1995) Expression of human beta-amyloid peptide in transgenic Caenorhabditis elegans. Proc Natl Acad Sci U S A 92: 9368–9372. 7568134 10.1073/pnas.92.20.9368 PMC40986

[39] Morley JF, Brignull HR, Weyers JJ, Morimoto RI (2002) The threshold for polyglutamine-expansion protein aggregation and cellular toxicity is dynamic and influenced by aging in Caenorhabditis elegans. Proc Natl Acad Sci U S A 99: 10417–10422. 12122205 10.1073/pnas.152161099 PMC124929

[40] Brignull HR, Moore FE, Tang SJ, Morimoto RI (2006) Polyglutamine proteins at the pathogenic threshold display neuron-specific aggregation in a pan-neuronal Caenorhabditis elegans model. J Neurosci 26: 7597–7606. 16855087 10.1523/JNEUROSCI.0990-06.2006 PMC6674286

[41] Gidalevitz T, Krupinski T, Garcia S, Morimoto RI (2009) Destabilizing protein polymorphisms in the genetic background direct phenotypic expression of mutant SOD1 toxicity. PLoS Genet 5: e1000399 doi:10.1371/journal.pgen.1000399. 19266020 10.1371/journal.pgen.1000399 PMC2642731

[42] van Ham TJ, Thijssen KL, Breitling R, Hofstra RM, Plasterk RH, et al (2008) C. elegans model identifies genetic modifiers of alpha-synuclein inclusion formation during aging. PLoS Genet 4: e1000027 doi:10.1371/journal.pgen.1000027. 18369446 10.1371/journal.pgen.1000027 PMC2265412

[43] Park KW, Li L (2008) Cytoplasmic expression of mouse prion protein causes severe toxicity in Caenorhabditis elegans. Biochem Biophys Res Commun 372: 697–702. 18519028 10.1016/j.bbrc.2008.05.132 PMC2587115

[44] Liu JJ, Lindquist S (1999) Oligopeptide-repeat expansions modulate ‘protein-only’ inheritance in yeast. Nature 400: 573–576. 10448860 10.1038/23048

[45] Melendez A, Talloczy Z, Seaman M, Eskelinen EL, Hall DH, et al (2003) Autophagy genes are essential for dauer development and life-span extension in C. elegans. Science 301: 1387–1391. 12958363 10.1126/science.1087782

[46] Ravikumar B, Imarisio S, Sarkar S, O'Kane CJ, Rubinsztein DC (2008) Rab5 modulates aggregation and toxicity of mutant huntingtin through macroautophagy in cell and fly models of Huntington disease. J Cell Sci 121: 1649–1660. 18430781 10.1242/jcs.025726 PMC2635563

[47] Liu Q, Chen B, Gaier E, Joshi J, Wang ZW (2006) Low conductance gap junctions mediate specific electrical coupling in body-wall muscle cells of Caenorhabditis elegans. J Biol Chem 281: 7881–7889. 16434400 10.1074/jbc.M512382200

[48] Shaner NC, Steinbach PA, Tsien RY (2005) A guide to choosing fluorescent proteins. Nat Methods 2: 905–909. 16299475 10.1038/nmeth819

[49] Grant BD, Sato M (2006) Intracellular trafficking. WormBook 1–9. 18050485 10.1895/wormbook.1.77.1 PMC4781316

[50] Grant B, Zhang Y, Paupard MC, Lin SX, Hall DH, et al (2001) Evidence that RME-1, a conserved C. elegans EH-domain protein, functions in endocytic recycling. Nat Cell Biol 3: 573–579. 11389442 10.1038/35078549

[51] Derkatch IL, Uptain SM, Outeiro TF, Krishnan R, Lindquist SL, et al (2004) Effects of Q/N-rich, polyQ, and non-polyQ amyloids on the de novo formation of the [PSI+] prion in yeast and aggregation of Sup35 in vitro. Proc Natl Acad Sci U S A 101: 12934–12939. 15326312 10.1073/pnas.0404968101 PMC516497

[52] Krammer C, Kryndushkin D, Suhre MH, Kremmer E, Hofmann A, et al (2009) The yeast Sup35NM domain propagates as a prion in mammalian cells. Proc Natl Acad Sci U S A 106: 462–467. 19114662 10.1073/pnas.0811571106 PMC2626725

[53] Habenstein B, Bousset L, Sourigues Y, Kabani M, Loquet A, et al (2012) A native-like conformation for the C-terminal domain of the prion Ure2p within its fibrillar form. Angew Chem Int Ed Engl 51: 7963–7966. 22760922 10.1002/anie.201202093

[54] Bousset L, Thomson NH, Radford SE, Melki R (2002) The yeast prion Ure2p retains its native alpha-helical conformation upon assembly into protein fibrils in vitro. EMBO J 21: 2903–2911. 12065404 10.1093/emboj/cdf303 PMC126058

[55] Gidalevitz T, Ben-Zvi A, Ho KH, Brignull HR, Morimoto RI (2006) Progressive disruption of cellular protein folding in models of polyglutamine diseases. Science 311: 1471–1474. 16469881 10.1126/science.1124514

[56] Mohri-Shiomi A, Garsin DA (2008) Insulin signaling and the heat shock response modulate protein homeostasis in the Caenorhabditis elegans intestine during infection. J Biol Chem 283: 194–201. 17951251 10.1074/jbc.M707956200

[57] Fevrier B, Vilette D, Archer F, Loew D, Faigle W, et al (2004) Cells release prions in association with exosomes. Proc Natl Acad Sci U S A 101: 9683–9688. 15210972 10.1073/pnas.0308413101 PMC470735

[58] Gousset K, Schiff E, Langevin C, Marijanovic Z, Caputo A, et al (2009) Prions hijack tunnelling nanotubes for intercellular spread. Nat Cell Biol 11: 328–336. 19198598 10.1038/ncb1841

[59] Garden GA, La Spada AR (2012) Intercellular (mis)communication in neurodegenerative disease. Neuron 73: 886–901. 22405200 10.1016/j.neuron.2012.02.017 PMC3334539

[60] Chiesa R, Piccardo P, Ghetti B, Harris DA (1998) Neurological illness in transgenic mice expressing a prion protein with an insertional mutation. Neuron 21: 1339–1351. 9883727 10.1016/s0896-6273(00)80653-4

[61] Flechsig E, Shmerling D, Hegyi I, Raeber AJ, Fischer M, et al (2000) Prion protein devoid of the octapeptide repeat region restores susceptibility to scrapie in PrP knockout mice. Neuron 27: 399–408. 10985358 10.1016/s0896-6273(00)00046-5

[62] McGlinchey RP, Kryndushkin D, Wickner RB (2011) Suicidal [PSI+] is a lethal yeast prion. Proc Natl Acad Sci U S A 108: 5337–5341. 21402947 10.1073/pnas.1102762108 PMC3069153

[63] Garcea RL, Schachat F, Epstein HF (1978) Coordinate synthesis of two myosins in wild-type and mutant nematode muscle during larval development. Cell 15: 421–428. 719748 10.1016/0092-8674(78)90011-9

[64] Tapley EC, Ly N, Starr DA (2011) Multiple mechanisms actively target the SUN protein UNC-84 to the inner nuclear membrane. Mol Biol Cell 22: 1739–1752. 21411627 10.1091/mbc.E10-08-0733 PMC3093325

[65] Hara T, Nakamura K, Matsui M, Yamamoto A, Nakahara Y, et al (2006) Suppression of basal autophagy in neural cells causes neurodegenerative disease in mice. Nature 441: 885–889. 16625204 10.1038/nature04724

[66] Komatsu M, Waguri S, Chiba T, Murata S, Iwata J, et al (2006) Loss of autophagy in the central nervous system causes neurodegeneration in mice. Nature 441: 880–884. 16625205 10.1038/nature04723

[67] Levine B, Kroemer G (2009) Autophagy in aging, disease and death: the true identity of a cell death impostor. Cell Death Differ 16: 1–2. 19079285 10.1038/cdd.2008.139 PMC2717606

[68] Martinez-Vicente M, Cuervo AM (2007) Autophagy and neurodegeneration: when the cleaning crew goes on strike. Lancet Neurol 6: 352–361. 17362839 10.1016/S1474-4422(07)70076-5

[69] Heiseke A, Aguib Y, Schatzl HM Autophagy, prion infection and their mutual interactions. Curr Issues Mol Biol 12: 87–97. 19767652

[70] Aguib Y, Heiseke A, Gilch S, Riemer C, Baier M, et al (2009) Autophagy induction by trehalose counteracts cellular prion infection. Autophagy 5: 361–369. 19182537 10.4161/auto.5.3.7662

[71] Heiseke A, Aguib Y, Riemer C, Baier M, Schatzl HM (2009) Lithium induces clearance of protease resistant prion protein in prion-infected cells by induction of autophagy. J Neurochem 109: 25–34. 19183256 10.1111/j.1471-4159.2009.05906.x

[72] Spencer B, Potkar R, Trejo M, Rockenstein E, Patrick C, et al (2009) Beclin 1 gene transfer activates autophagy and ameliorates the neurodegenerative pathology in alpha-synuclein models of Parkinson's and Lewy body diseases. J Neurosci 29: 13578–13588. 19864570 10.1523/JNEUROSCI.4390-09.2009 PMC2812014

[73] Kruger U, Wang Y, Kumar S, Mandelkow EM (2011) Autophagic degradation of tau in primary neurons and its enhancement by trehalose. Neurobiol Aging 10.1016/j.neurobiolaging.2011.11.009 22169203

[74] Schaeffer V, Lavenir I, Ozcelik S, Tolnay M, Winkler DT, et al (2012) Stimulation of autophagy reduces neurodegeneration in a mouse model of human tauopathy. Brain 10.1093/brain/aws143 PMC3381726 22689910

[75] Chen SF, Kang ML, Chen YC, Tang HW, Huang CW, et al (2012) Autophagy-related gene 7 is downstream of heat shock protein 27 in the regulation of eye morphology, polyglutamine toxicity, and lifespan in Drosophila. J Biomed Sci 19: 52. 22621211 10.1186/1423-0127-19-52 PMC3483682

[76] Lee JA, Gao FB (2009) Inhibition of autophagy induction delays neuronal cell loss caused by dysfunctional ESCRT-III in frontotemporal dementia. J Neurosci 29: 8506–8511. 19571141 10.1523/JNEUROSCI.0924-09.2009 PMC2726650

[77] Charroux B, Fanto M (2010) The fine line between waste disposal and recycling: DRPLA fly models illustrate the importance of completing the autophagy cycle for rescuing neurodegeneration. Autophagy 6 10.4161/auto.6.5.12433 20543566

[78] Ling D, Salvaterra PM (2011) Brain aging and Abeta(1)(-)(4)(2) neurotoxicity converge via deterioration in autophagy-lysosomal system: a conditional Drosophila model linking Alzheimer's neurodegeneration with aging. Acta Neuropathol 121: 183–191. 21076961 10.1007/s00401-010-0772-0

[79] Dehay B, Bove J, Rodriguez-Muela N, Perier C, Recasens A, et al (2010) Pathogenic lysosomal depletion in Parkinson's disease. J Neurosci 30: 12535–12544. 20844148 10.1523/JNEUROSCI.1920-10.2010 PMC6633458

[80] Bossy-Wetzel E, Barsoum MJ, Godzik A, Schwarzenbacher R, Lipton SA (2003) Mitochondrial fission in apoptosis, neurodegeneration and aging. Curr Opin Cell Biol 15: 706–716. 14644195 10.1016/j.ceb.2003.10.015

[81] Settembre C, Fraldi A, Jahreiss L, Spampanato C, Venturi C, et al (2008) A block of autophagy in lysosomal storage disorders. Hum Mol Genet 17: 119–129. 17913701 10.1093/hmg/ddm289

[82] Settembre C, Fraldi A, Rubinsztein DC, Ballabio A (2008) Lysosomal storage diseases as disorders of autophagy. Autophagy 4: 113–114. 18000397 10.4161/auto.5227

[83] de Pablo-Latorre R, Saide A, Polishhuck EV, Nusco E, Fraldi A, et al (2012) Impaired parkin-mediated mitochondrial targeting to autophagosomes differentially contributes to tissue pathology in lysosomal storage diseases. Hum Mol Genet 21: 1770–1781. 22215441 10.1093/hmg/ddr610 PMC3313794

[84] Jaiswal JK, Andrews NW, Simon SM (2002) Membrane proximal lysosomes are the major vesicles responsible for calcium-dependent exocytosis in nonsecretory cells. J Cell Biol 159: 625–635. 12438417 10.1083/jcb.200208154 PMC2173094

[85] Reddy A, Caler EV, Andrews NW (2001) Plasma membrane repair is mediated by Ca(2+)-regulated exocytosis of lysosomes. Cell 106: 157–169. 11511344 10.1016/s0092-8674(01)00421-4

[86] Yasuda K, Khandare A, Burianovskyy L, Maruyama S, Zhang F, et al (2011) Tunneling nanotubes mediate rescue of prematurely senescent endothelial cells by endothelial progenitors: exchange of lysosomal pool. Aging (Albany NY) 3: 597–608. 21705809 10.18632/aging.100341 PMC3164368

[87] Terman A, Brunk UT (1998) Ceroid/lipofuscin formation in cultured human fibroblasts: the role of oxidative stress and lysosomal proteolysis. Mech Ageing Dev 104: 277–291. 9818731 10.1016/s0047-6374(98)00073-6

[88] Krammer C, Suhre MH, Kremmer E, Diemer C, Hess S, et al (2008) Prion protein/protein interactions: fusion with yeast Sup35p-NM modulates cytosolic PrP aggregation in mammalian cells. FASEB J 22: 762–773. 17928365 10.1096/fj.07-8733com

[89] Garrity SJ, Sivanathan V, Dong J, Lindquist S, Hochschild A (2010) Conversion of a yeast prion protein to an infectious form in bacteria. Proc Natl Acad Sci U S A 107: 10596–10601. 20484678 10.1073/pnas.0913280107 PMC2890818

[90] Derkatch IL, Bradley ME, Hong JY, Liebman SW (2001) Prions affect the appearance of other prions: the story of [PIN(+)]. Cell 106: 171–182. 11511345 10.1016/s0092-8674(01)00427-5

[91] Derkatch IL, Bradley ME, Zhou P, Chernoff YO, Liebman SW (1997) Genetic and environmental factors affecting the de novo appearance of the [PSI+] prion in Saccharomyces cerevisiae. Genetics 147: 507–519. 9335589 10.1093/genetics/147.2.507 PMC1208174

[92] Osherovich LZ, Weissman JS (2001) Multiple Gln/Asn-rich prion domains confer susceptibility to induction of the yeast [PSI(+)] prion. Cell 106: 183–194. 11511346 10.1016/s0092-8674(01)00440-8

[93] Michelitsch MD, Weissman JS (2000) A census of glutamine/asparagine-rich regions: implications for their conserved function and the prediction of novel prions. Proc Natl Acad Sci U S A 97: 11910–11915. 11050225 10.1073/pnas.97.22.11910 PMC17268

[94] Chen B, Bruce KL, Newnam GP, Gyoneva S, Romanyuk AV, et al (2010) Genetic and epigenetic control of the efficiency and fidelity of cross-species prion transmission. Mol Microbiol 76: 1483–1499. 20444092 10.1111/j.1365-2958.2010.07177.x PMC3025758

[95] Chien P, DePace AH, Collins SR, Weissman JS (2003) Generation of prion transmission barriers by mutational control of amyloid conformations. Nature 424: 948–951. 12931190 10.1038/nature01894

[96] Fire A, Harrison SW, Dixon D (1990) A modular set of lacZ fusion vectors for studying gene expression in Caenorhabditis elegans. Gene 93: 189–198. 2121610 10.1016/0378-1119(90)90224-f

[97] Brenner S (1974) The genetics of Caenorhabditis elegans. Genetics 77: 71–94. 4366476 10.1093/genetics/77.1.71 PMC1213120

[98] Krzewska J, Tanaka M, Burston SG, Melki R (2007) Biochemical and functional analysis of the assembly of full-length Sup35p and its prion-forming domain. J Biol Chem 282: 1679–1686. 17121860 10.1074/jbc.M608110200

[99] Walsh DM, Thulin E, Minogue AM, Gustavsson N, Pang E, et al (2009) A facile method for expression and purification of the Alzheimer's disease-associated amyloid beta-peptide. FEBS J 276: 1266–1281. 19175671 10.1111/j.1742-4658.2008.06862.x PMC2702495

[100] Thual C, Komar AA, Bousset L, Fernandez-Bellot E, Cullin C, et al (1999) Structural characterization of Saccharomyces cerevisiae prion-like protein Ure2. J Biol Chem 274: 13666–13674. 10224139 10.1074/jbc.274.19.13666

