# Supplementary figures and images for: Spreading of a Prion Domain from Cell-to-Cell by Vesicular Transport in Caenorhabditis elegans
Source: PLoS Genet. 2013 Mar 28;9(3):e1003351. doi: 10.1371/journal.pgen.1003351 (PMC3610634; doi:10.1371/journal.pgen.1003351)

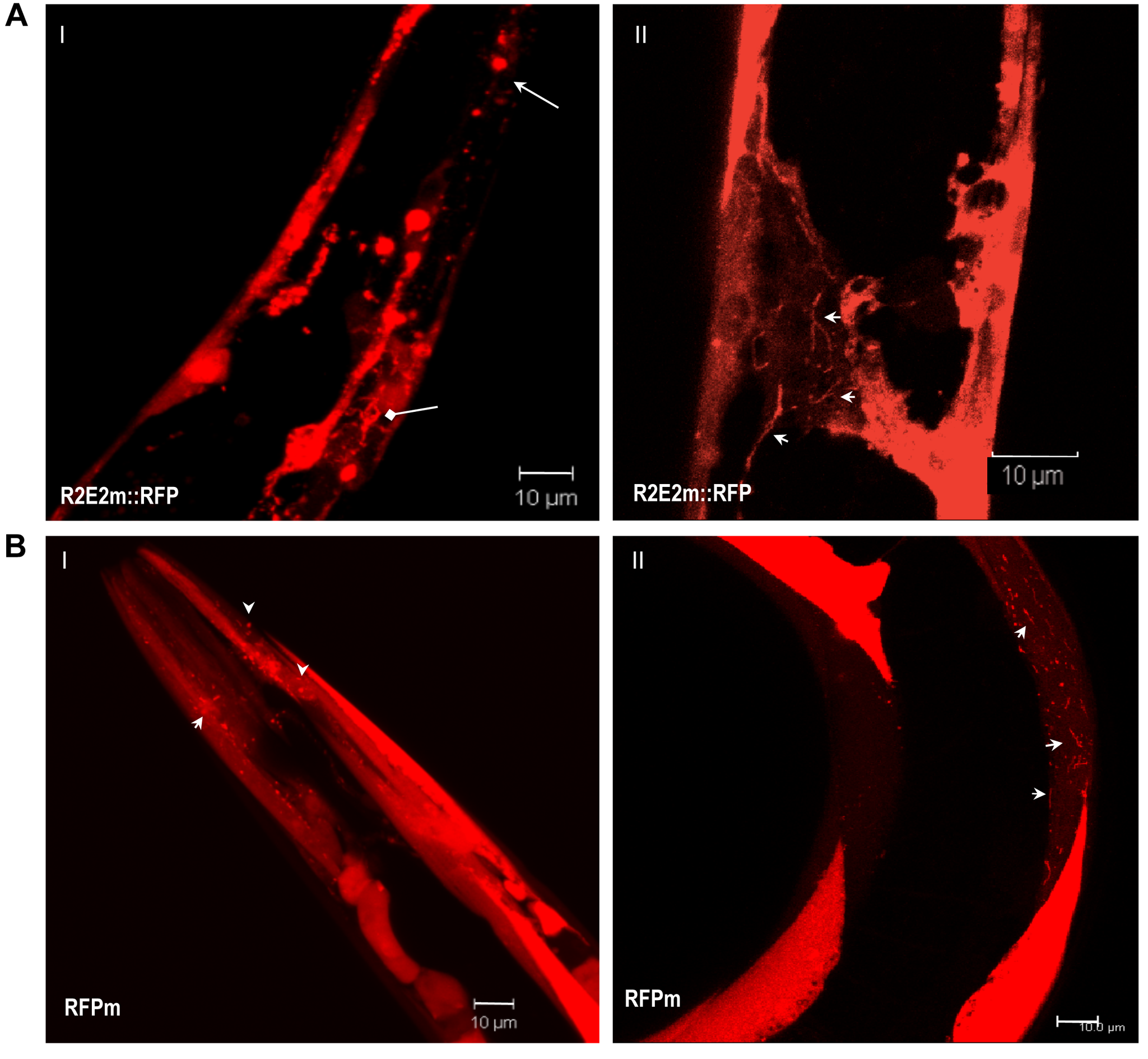

Supplement: Figure S1 — Detailed description of aggregates and vesicular structures occuring in R2E2m::RFP and RFPm expressing animals. (A) Collapsed confocal z-stack images of R2E2m::RFP expressing nematodes reveal tubular vesicles in addition to multiple types of aggregate structures. (B) The RFP tag alone remains mostly soluble upon expression in BWM cells, but was also occasionally found in vesicular structures and small aggregates. The majority of these vesicles remained stationary (data not shown), indicating that although RFP is able to visualize acidic vesicles, the occurrence of moving tubular vesicles is associated with the aggregation-prone prion domain. Open arrow and diamond arrow point to large round, and fibril-like aggregates, respectively. Arrowheads indicate small aggregates (indistinguishable from small round vesicles) or round and tubular vesicles. (TIF) [file pgen.1003351.s001.tif]

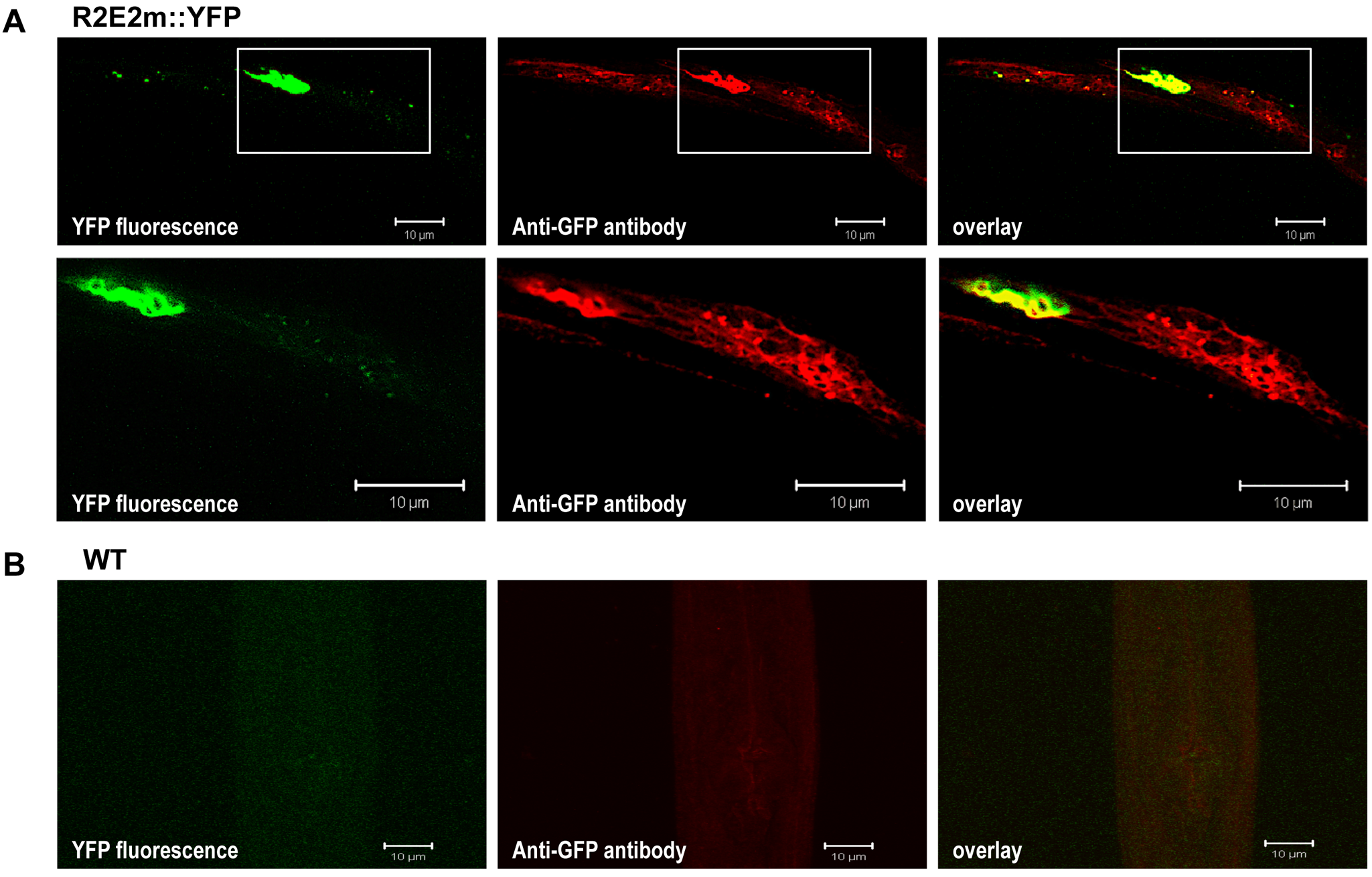

Supplement: Figure S2 — R2E2m::YFP does form vesicular structures that can be visualized by indirect immunofluorescence. (A) R2E2m::YFP stained with anti-GFP antibody. The GFP staining reveals vesicular structures that are not visible with YFP fluorescence. The fibril-like aggregate was overexposed in both channels (YFP and RFP) to better show vesicular structures in RFP and to demonstrate the absence of YFP-positive vesicular structures. (B) N2 wild-type control stained with anti-GFP antibody. No unspecific staining of the anti-GFP antibody was observed. Scale bars: 10 µm. (TIF) [file pgen.1003351.s002.tif]

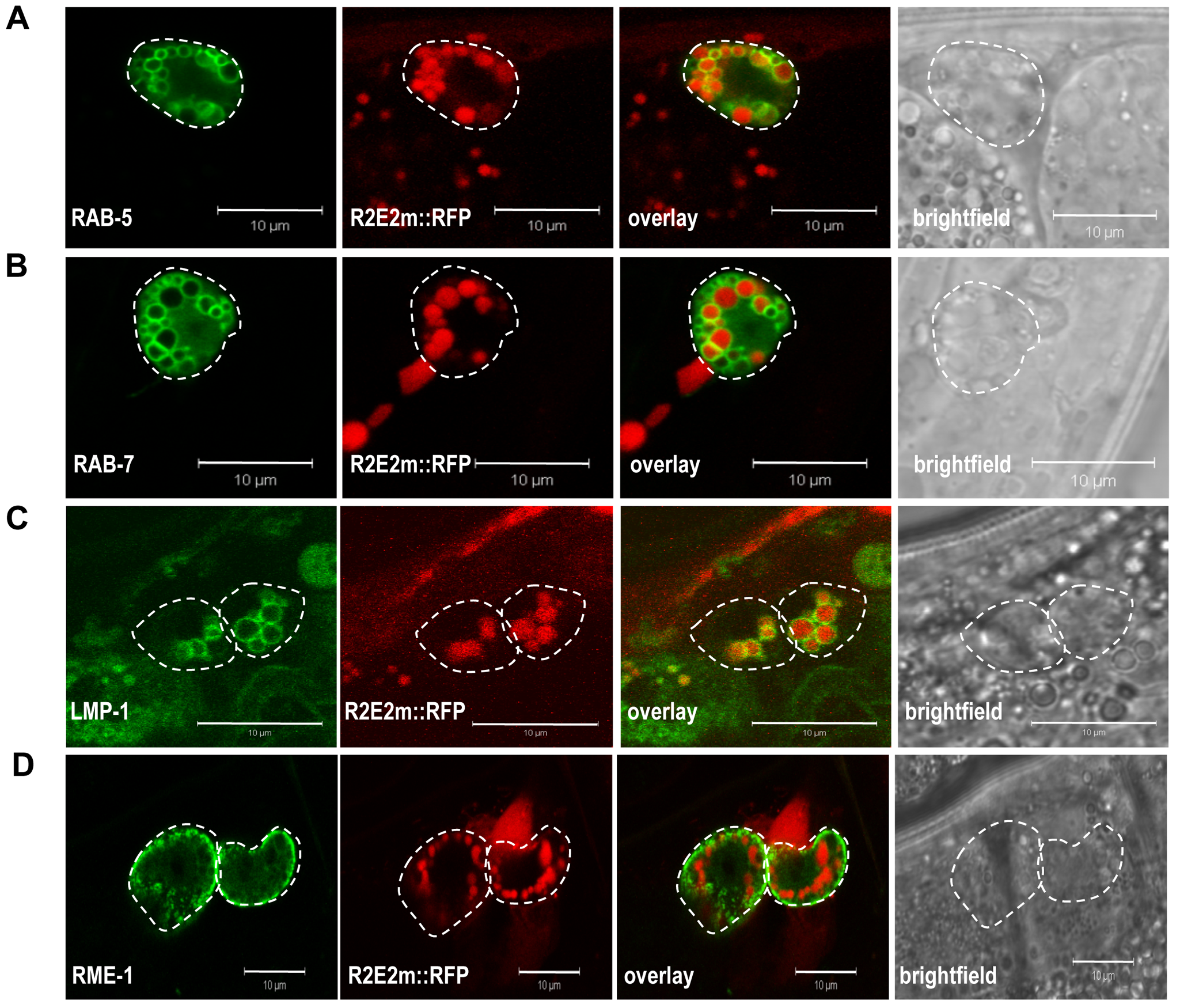

Supplement: Figure S3 — R2E2m::RFP gets released into the body cavity and endocytosed by coelomocytes. Confocal fluorescence and brightfield images of animals carrying the indicated coelomocyte vesicle markers and expressing R2E2m::RFP in BWM cells. Coelomocytes are outlined. R2E2m::RFP does co-localize with (A) early (RAB-5) and (B) late (RAB-7) endosomes, and (C) lysosomes (LMP-1), but not with (D) recycling endosomes (RME-1). Scale bars: 10 µm. (TIF) [file pgen.1003351.s003.tif]

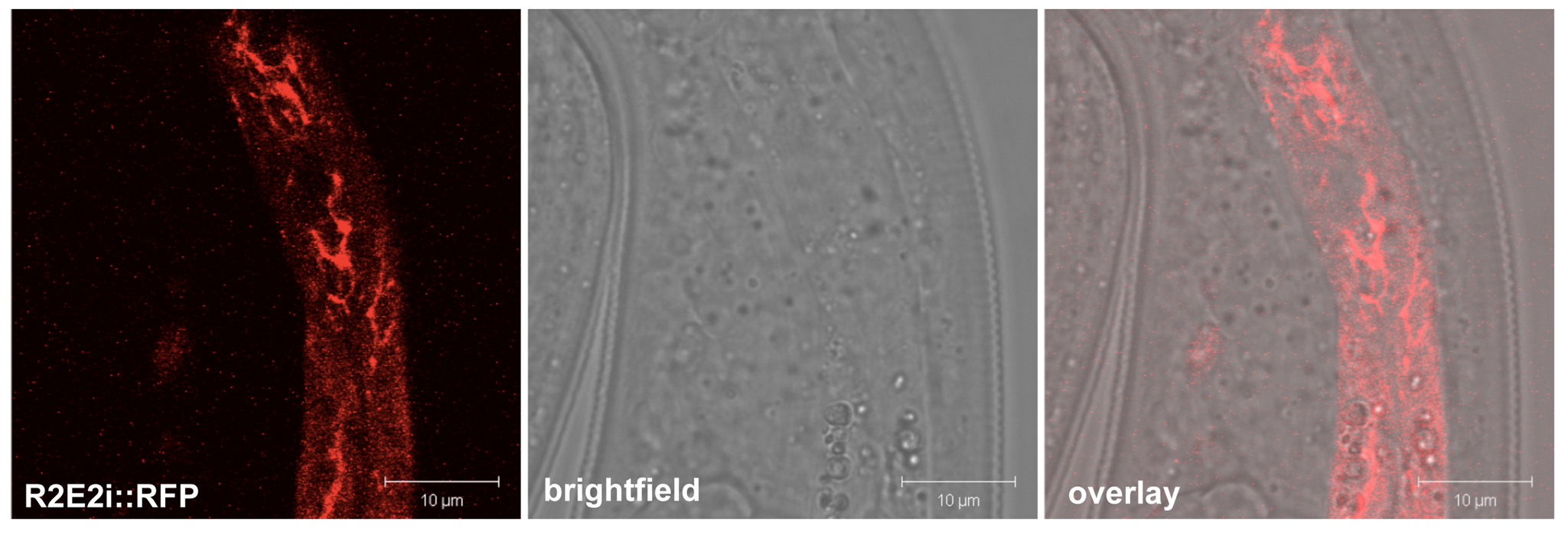

Supplement: Figure S4 — R2E2i::RFP containing vesicles emerge also in C. elegans intestinal cells. Confocal fluorescence and brightfield images of animals expressing R2E2i::RFP in the intestine. Scale bar: 10 µm. (TIF) [file pgen.1003351.s004.tif]

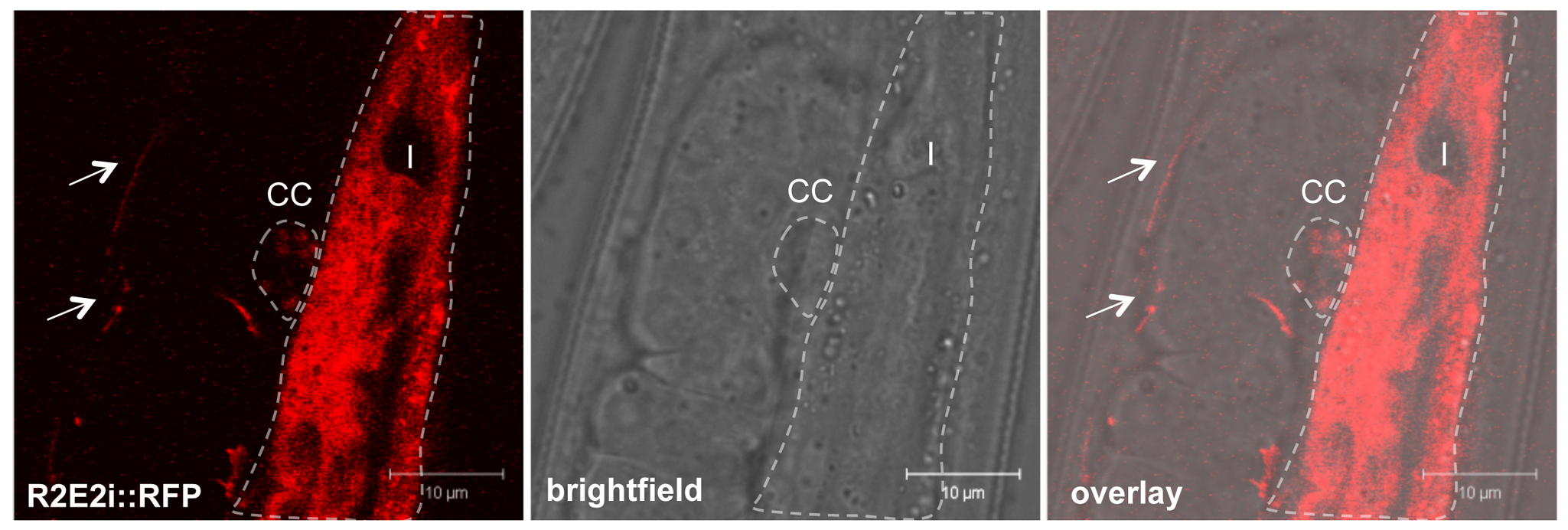

Supplement: Figure S5 — R2E2i::RFP containing vesicles are able to exit from C. elegans intestinal cells and spread into non-expressing tissues. Confocal fluorescence and brightfield images of animals expressing R2E2i::RFP in intestinal cells. Intestine (I) and coelomocytes (CC) are outlined. Arrows indicate distant vesicles that have been release from the intestine. As the low fluorescence of released vesicles reaches the detection limit of the microscope, the RFP channel was overexposed to better show vesicular structures outside of the intestine. Scale bar: 10 µm. (TIF) [file pgen.1003351.s005.tif]

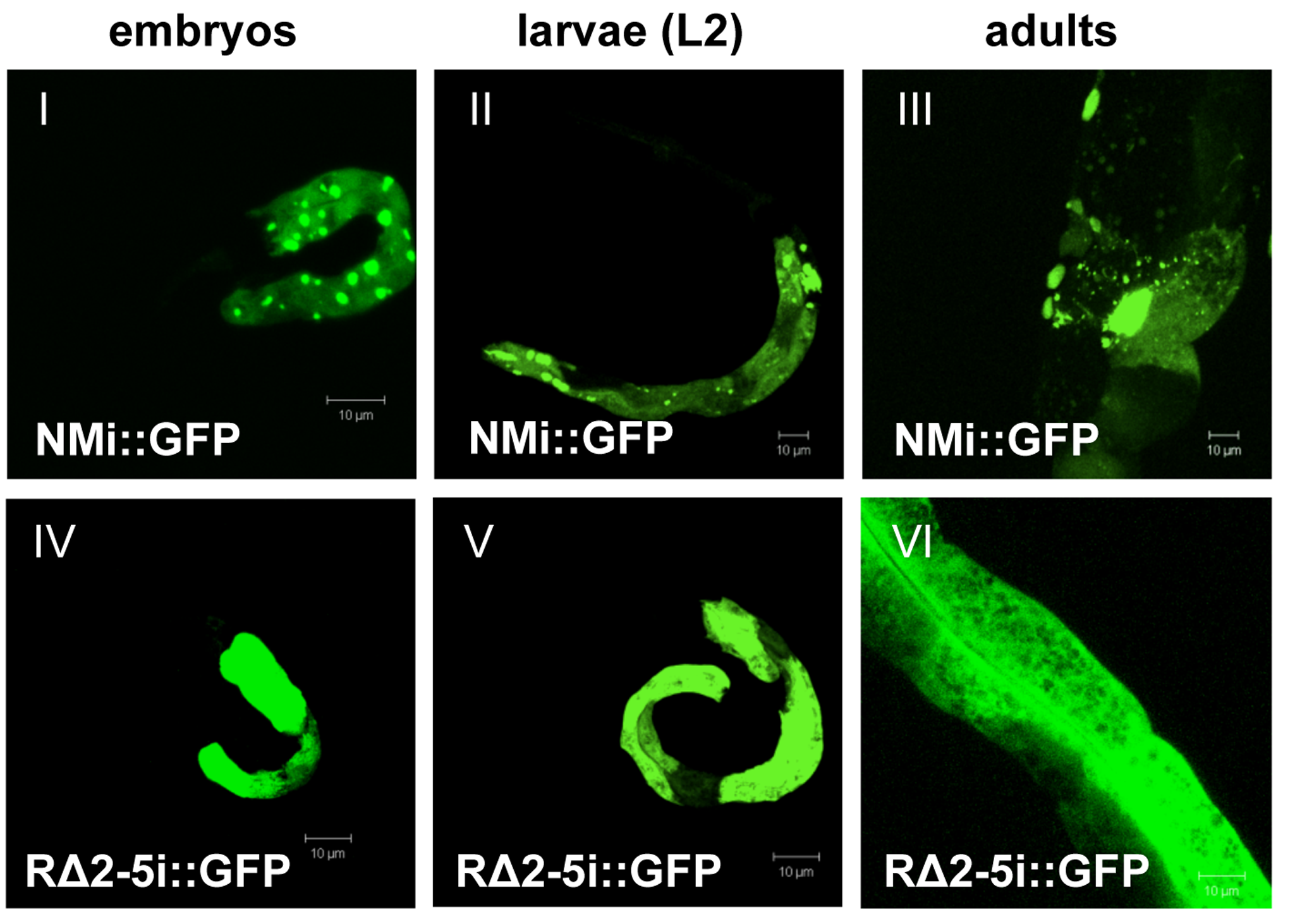

Supplement: Figure S6 — Oligopeptide repeat region dependent aggregation of the prion domain in intestinal cells. Collapsed confocal z-stack images of C. elegans lines stably expressing the indicated transgene. Pictures were taken at displayed stages during nematode development (embryo/L2 larvae/adult). Scale bar: 10 µm. (TIF) [file pgen.1003351.s006.tif]

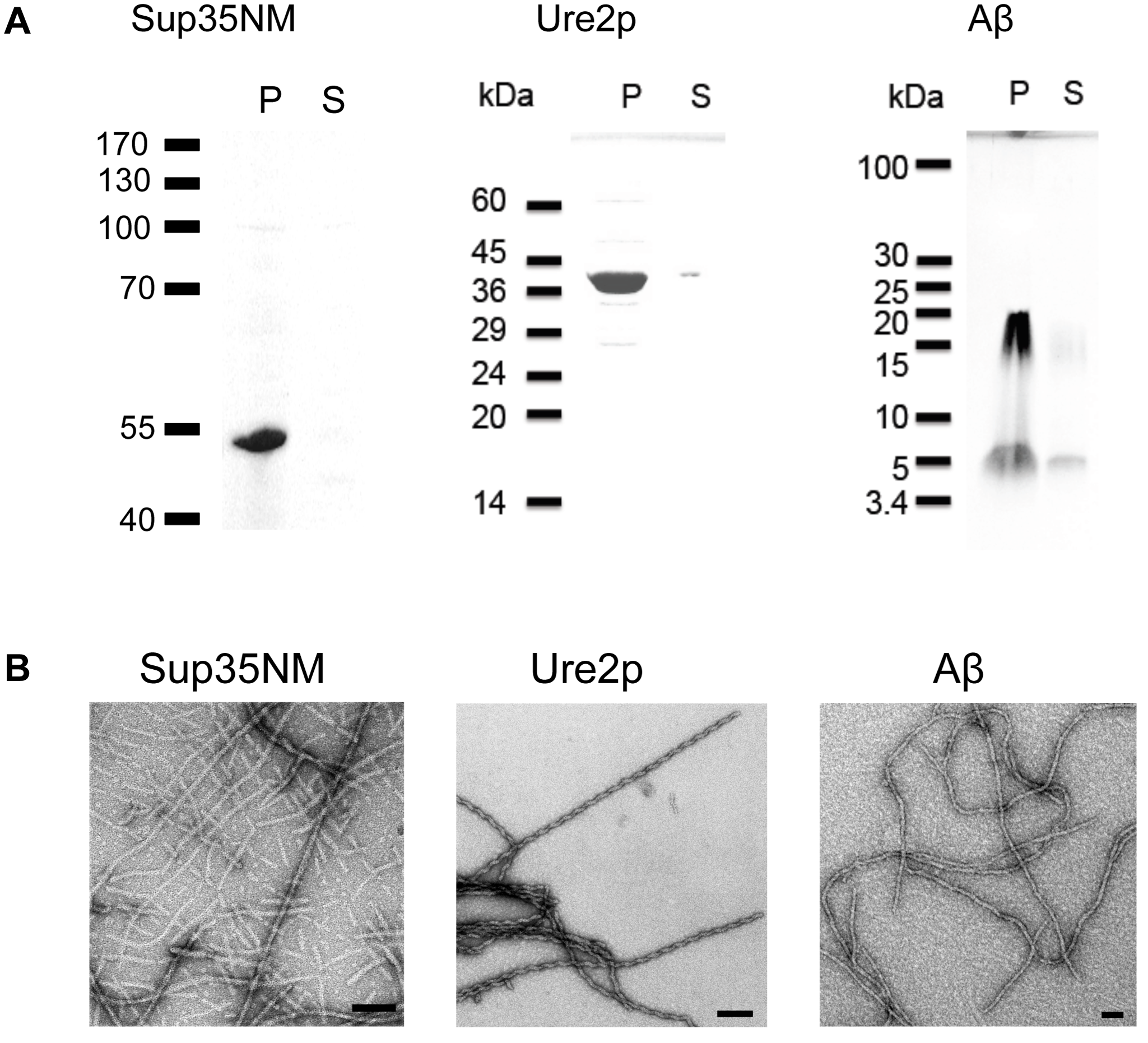

Supplement: Figure S7 — Quality control of fibrilized proteins used for microinjection. SDS solubility assays (A) and TEM images (B) of indicated fibrilized proteins. P = pellet, S = supernatant. Scale bars: 0.1 µm in the panels Sup35NM and Ure2p, 0.01 µm in the panel Aβ. (TIF) [file pgen.1003351.s007.tif]

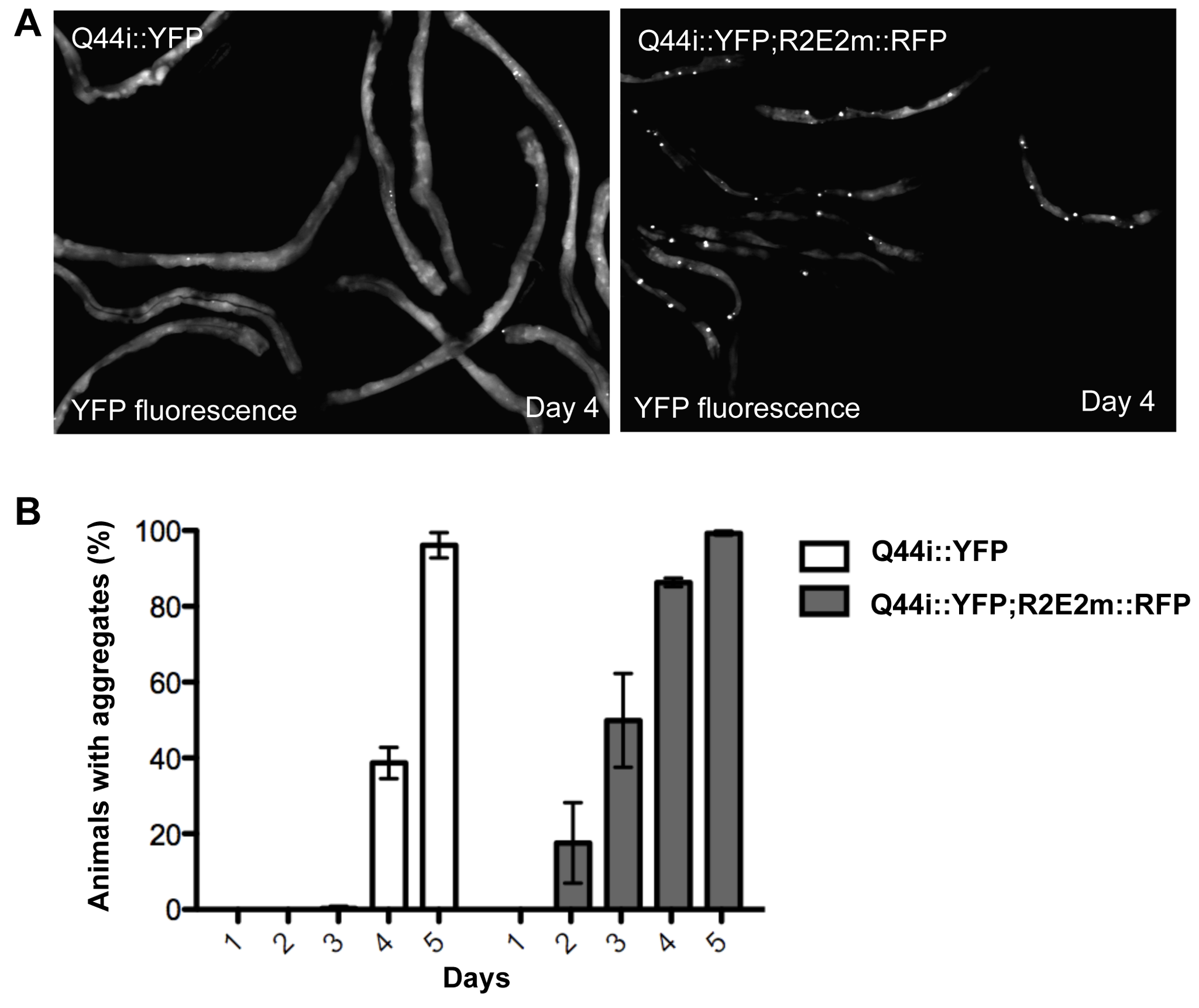

Supplement: Figure S8 — R2E2m::RFP induces cell non-autonomous aggregation of Q44i::YFP. (A). Representative fluorescent images of Q44i::YFP and Q44i::YFP;R2E2m::RFP animals on day 4 after transferring synchronized L1 larvae onto fresh OP50-seeded NGM plates. The expression of R2E2 in BWM cells led to an earlier onset of Q44 aggregation in the intestine. (B). Quantification of animals with aggregates (in %) on indicated days after synchronization. Error bars: S.D. (TIF) [file pgen.1003351.s008.tif]
